# Tumor-associated neutrophils upregulate Nectin2 expression, creating the immunosuppressive microenvironment in pancreatic ductal adenocarcinoma

**DOI:** 10.1186/s13046-024-03178-6

**Published:** 2024-09-11

**Authors:** Haizhen Luo, Naoki Ikenaga, Kohei Nakata, Nobuhiro Higashijima, Pingshan Zhong, Akihiro Kubo, Chenyi Wu, Chikanori Tsutsumi, Yuki Shimada, Masataka Hayashi, Koki Oyama, Satomi Date, Toshiya Abe, Noboru Ideno, Chika Iwamoto, Koji Shindo, Kenoki Ohuchida, Yoshinao Oda, Masafumi Nakamura

**Affiliations:** 1 Department of Surgery and Oncology, Graduate School of Medical Sciences, Kyushu University, 3-1-1 Maidashi, Higashi-Ku, Fukuoka, 812-8582 Japan; 2 Department of Endoscopic Diagnostics and Therapeutics, Kyushu University Hospital, Fukuoka, 812- 8582 Japan; 3 Department of International Medicine, Kyushu University Hospital, Fukuoka, 812-8582 Japan; 4 Department of Anatomical Pathology, Graduate School of Medical Sciences, Kyushu University, 3-1-1 Maidashi, Higashi-Ku, Fukuoka, 812-8582 Japan; 5 Department of Pathology, The Sol Goldman Pancreatic Cancer Research Center, Johns Hopkins Medical Institutions, Baltimore, MD USA

**Keywords:** Nectin2, Tumor-associated neutrophils, Chemokine ligand 5, Pancreatic cancer, Tumor immune microenvironment

## Abstract

**Background:**

Tumor-associated neutrophils (TANs) constitute an abundant component among tumor-infiltrating immune cells and have recently emerged as a critical player in pancreatic ductal adenocarcinoma (PDAC) progression. This study aimed to elucidate the pro-tumor mechanisms of TAN and identify a novel target for effective immunotherapy against PDAC.

**Methods:**

Microarray and cytokine array analyses were performed to identify the mechanisms underlying the function of TANs. Human and mouse TANs were obtained from differentiated HL-60 cells and orthotopically transplanted PDAC tumors, respectively. The interactions of TANs with cancer and cytotoxic T-cells were evaluated through in vitro co-culture and in vivo orthotopic or subcutaneous models. Single-cell transcriptomes from patients with PDAC were analyzed to validate the cellular findings.

**Results:**

Increased neutrophil infiltration in the tumor microenvironment was associated with poor survival in patients with PDAC. TANs secreted abundant amounts of chemokine ligand 5 (CCL5), subsequently enhancing cancer cell migration and invasion. TANs subpopulations negatively correlated with cytotoxic CD8^+^ T-cell infiltration in PDAC and promoted T-cell dysfunction. TANs upregulated the membranous expression of Nectin2, which contributed to CD8^+^ T-cell exhaustion. Blocking Nectin2 improved CD8^+^ T-cell function and suppressed tumor progression in the mouse model. Single-cell analysis of human PDAC revealed two immunosuppressive TANs phenotypes: Nectin2^+^ TANs and OLR1^+^ TANs. Endoplasmic reticulum stress regulated the protumor activities in TANs.

**Conclusions:**

TANs enhance PDAC progression by secreting CCL5 and upregulating Nectin2. Targeting the immune checkpoint Nectin2 could represent a novel strategy to enhance immunotherapy efficacy in PDAC.

**Supplementary Information:**

The online version contains supplementary material available at 10.1186/s13046-024-03178-6.

## Background

Pancreatic ductal adenocarcinoma (PDAC) has a dismal five-year survival rate of approximately 13% [1]. Immunosuppressive myeloid and T-regulatory cells within the tumor microenvironment (TME) substantially hinder antitumor immune responses in PDAC [2]. The intricate interactions among immune cells in the pancreatic TME contribute to aggressive disease progression and therapeutic resistance to current immune checkpoint inhibitors [3]. Therefore, elucidating these cellular interactions in the immunosuppressive TME is crucial for devising effective PDAC treatments.

Neutrophils constitute a considerable proportion of immune cells in the TME, and elevated neutrophil infiltration is associated with a poor prognosis in most solid tumors [4, 5]. They are functionally plastic and play multifaceted roles in solid cancer progression, influenced by surrounding environments [5]. While some neutrophils can combat cancer cells by releasing reactive oxygen species (ROS) and neutrophil elastase [6, 7], most tumor-associated neutrophils (TANs) are polarized by mediators from the TME, exhibiting tumor-promoting activities [8]. TANs enhance tumor progression by promoting cancer cell proliferation, angiogenesis, and tumor metastasis through protumor molecule secretion [9]. Moreover, TANs create an immunosuppressive TME by interacting with macrophages, natural killer (NK) cells, and T-cells [5, 9, 10] and inhibit cytotoxic T-cell function by producing ROS [11] and arginase 1 [12]. Additionally, a subpopulation of TANs expresses ligands for lymphocyte checkpoints such as PD1 ligand 1 (PD-L1) or 2 (PD-L2), inhibiting T-cell immunity through checkpoint pathways in patients with gastric and liver cancers [13–15]. These studies highlight the crucial role of TANs in tumor progression and indicate that targeting the protumor functions of TANs, including immunosuppressive activities, represents a novel frontier in PDAC treatment.

The advent of single-cell analysis has provided insights into the landscape and dynamics of immune cell populations within cancer tissues [16, 17]. A previous study using microwell-based single-cell RNA sequencing revealed four distinct subpopulations of TANs in PDAC. Moreover, it identified BHLHE40 as a novel transcriptional regulator promoting the polarization of infiltrating neutrophils toward an immunosuppressive phenotype [18]. This study provided the first comprehensive overview of TANs in PDAC. However, most results were based on a multi-omics approach, and a comprehensive mechanical evaluation of TANs was lacking. Therefore, a detailed functional characterization of TANs in PDAC is required to eliminate the immunosuppressive TME associated with this disease.

In this study, we identified novel molecules associated with protumor TANs, chemokine ligand 5 (CCL5) and Nectin2, and clarified their role in fostering an immunosuppressive TME. CCL5 secreted from TANs enhances the recruitment of regulatory T (Treg) cells into the TME and promotes cancer cells’ migration and invasion. The membranous expression of Nectin2 upregulated in TANs promotes CD8 + T-cell exhaustion as an immune checkpoint ligand. Our findings provide us with a novel strategy for improving immunotherapeutic approaches for PDAC by targeting TAN-specific molecules.

## Methods

### Human pancreatic ductal adenocarcinoma tissue samples and clinical data

The human PDAC tissue samples were obtained from patients who underwent pancreatic resection for PDAC at Kyushu University Hospital (Fukuoka, Japan) between 2012 and 2020, excluding those exhibiting positive intraoperative ascites cytology, noncancerous death, or acinar cell carcinoma during pathological diagnosis. Peripheral blood (PB) lymphocyte and neutrophil counts were obtained from blood tests performed within seven days before pancreatectomy. Additionally, normal pancreatic tissues were obtained from non-tumor areas of patients with resected pancreatic neuroendocrine tumors.

All patients provided informed consent, and the study adhered to the Declaration of Helsinki. The study protocol was approved by the Ethics Committee of Kyushu University (30–230; 858-00). Clinical data, including blood sample analyses and histopathological findings, were obtained from electronic medical records.

### Cell lines

We purchased HL-60, SUIT-2, and MIAPACA-2 cell lines from the Health Science Research Resources Bank (Japan). As described previously, murine PDAC cell lines (KPC-1 and KPC-2) were established from LSL-Kras^G12D/+^, LSL-Trp53^R172H/+^, and Pdx-1-Cre (KPC) mice [19]. SUIT-2, MIAPACA-2, and murine PDAC cell lines were maintained in Dulbecco’s modified Eagle’s medium (DMEM; Sigma-Aldrich, St. Louis, MO, USA) supplemented with 10% fetal bovine serum, streptomycin (100 mg/mL), and penicillin (100 mg/mL) at 37 °C in a humidified atmosphere containing 10% CO_2_.

### HL-60 derived neutrophils and their treatment

Differentiated neutrophils from HL-60 cells were induced through supplementation with 1.25% dimethyl sulfoxide and 1 µmol/L all-trans-retinoic acid for three days in Roswell Park Memorial Institute 1640 medium supplemented with 10% fetal bovine serum, streptomycin (100 mg/mL), and penicillin (100 mg/mL) at 37 °C in a humidified atmosphere with 5% CO_2_ [20]. HL-60 differentiation was confirmed by the appearance of typical segmented nuclei, and CD11b^+^CD14^−^ expression using a FACSAria flow cytometer (BD Biosciences, Franklin Lakes, NJ, USA). Differentiated neutrophils were stimulated with the supernatant of cancer cell line, SUIT-2 or MIAPACA-2 for 12 h to induce TANs. To inhibit ER stress in TANs, we pretreated TANs with 10 μm 4-PBA (P21005, Sigma) for 40 min before supplementation with the supernatant of cancer cell line.

### Transcriptome analysis (microarray analysis)

Total RNA was isolated from neutrophils using a High Pure RNA Isolation Kit (Roche) with DNase I (Roche). RNA quality was assessed through microarray analysis using an Agilent 2200 TapeStation (Agilent Technologies). Gene expression levels were determined using a SurePrint G3 Human GE Microarray8 × 60 K v3.0 (Agilent Technologies) according to the raw signal. All data were analyzed using Feature Extraction software (Agilent Technologies).

### Cytokine array

Cytokines were detected using a human cytokine antibody array (120 targets; AAH-CYT-1000; Raybio) and a mouse cytokine antibody array (120 targets; AAM-CYT-1000; Raybio) according to the manufacturer’s instructions. The supernatant (1.5 mL) was collected from neutrophils after 48-h culture and filtered through a sterile 0.22 μm filter (Millipore, Sigma) to remove cells. Images were acquired using ChemiDoc XRS (Bio-Rad Laboratories).

### Matrigel invasion and migration assays

For the migration assay, pancreatic cancer cells were seeded in Transwell chambers with 8 μm pores (#353097; BD Bioscience) at 5 × 10^4^ cells/well. For the invasion assay, Transwell chambers with 8 μm pores were coated with 20 µg/well Matrigel (#354234; BD Biosciences). To evaluate CCL5 function in these assays, a CCL5 neutralizing antibody (mouse#MAB478; human#MAB278; R&D Systems) was added in a lower chamber at 0.5 µg/mL. After 24 h, the chambers were placed into 24-well plates (#353504; Corning) with 750 µL neutrophil conditioned medium containing 10% FBS. Upper pancreatic cancer cells were suspended in 250 µL DMEM. After 24 (migration assay) or 48 h (invasion assay) of incubation, a cotton swab was used to remove non-invading cells, and the upper chamber was fixed with 70% ethanol. Cells were washed, stained with H&E, and counted in five random fields at 100× magnification using a BZ-X Analyzer (KEYENCE).

### Cell immunofluorescence staining

Neutrophils were plated in poly-Lysine coated-glass-bottomed dishes (#D11131H, Matsunami) at 1 × 10^6^ cells/well and fixed with − 20 °C ethanol. They were subsequently blocked with 3% BSA in PBS and incubated with anti-LC3 (1:100, #2775, Cell Signaling Technology) at 4 °C overnight. The corresponding secondary antibodies carrying a green fluorescent dye and 1 mg/mL nuclear DNA-binding DAPI were used. Samples were then incubated at room temperature for 60 min, washed with 0.1% BSA, and neutrophils were detected using a fluorescence microscope with BZ-X Analyzer software.

### Statistics

All statistical analyses and graphs were generated using GraphPad Prism (version 9.3.1, GraphPad, CA, USA). Comparisons between groups were performed using the Mann–Whitney test, unpaired Student’s t-test, or one-way analysis of variance. Survival analyses were conducted using the Kaplan–Meier method, and curves were compared using the log-rank test. Correlation analyses were conducted using Pearson’s correlation coefficients. Data are presented as the mean ± standard deviation. A p-value of < 0.05 was considered statistically significant. Additional materials and methods are described in the Supplementary Materials.

## Results

### Increased TANs infiltration in TME implicates PDAC progression

**Fig. 1:**
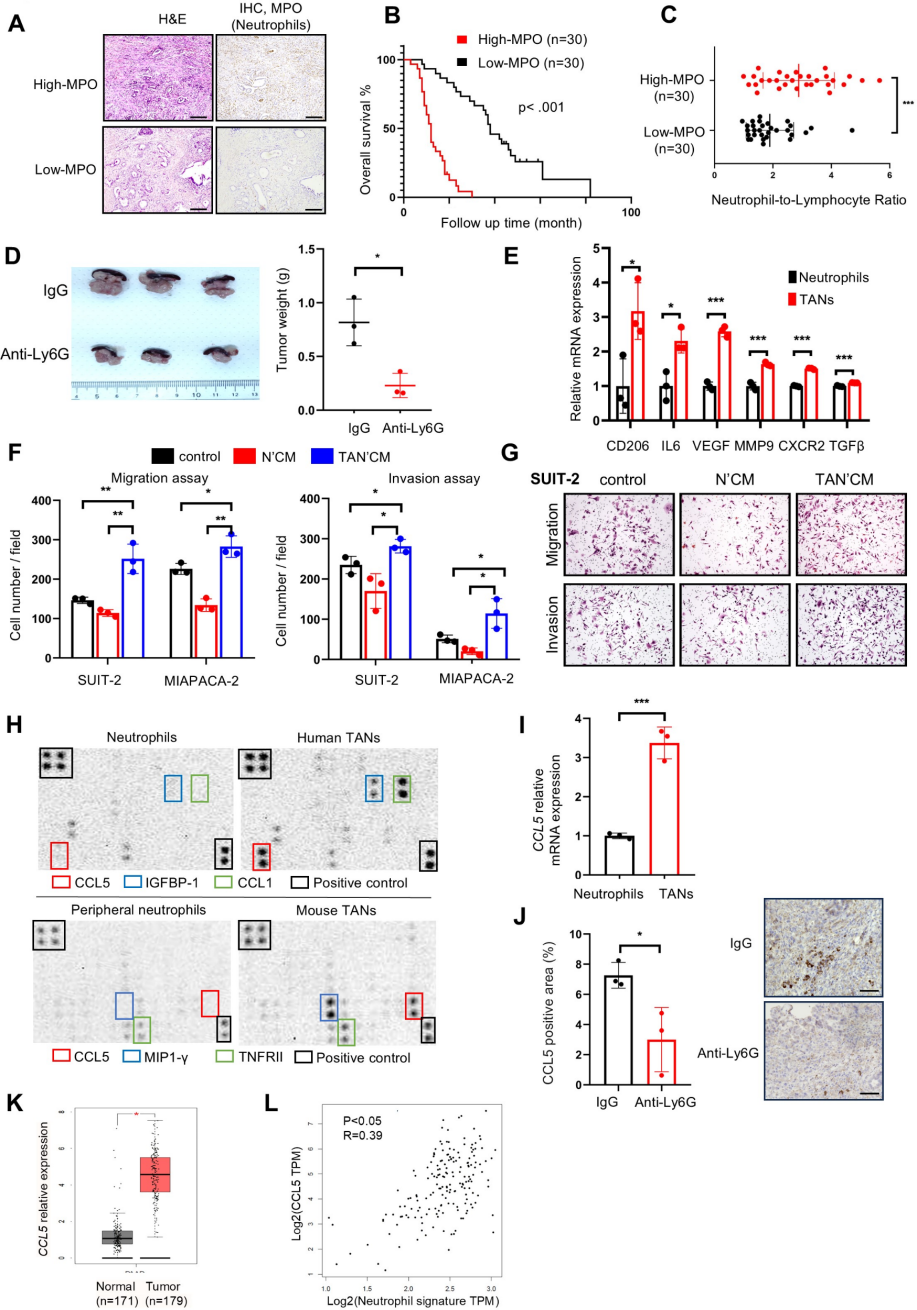
Tumor-associated neutrophils (TANs) infiltration is implicated in poor prognosis in pancreatic ductal adenocarcinoma (PDAC) and abundantly secretes CCL5 through polarization. (**A**) Representative images of H&E and immunohistochemistry of myeloperoxidase (MPO) in human PDAC. High- (top) and low-MPO (bottom) indicate abundant and scarce infiltration of neutrophils in the tumor microenvironment (TME), respectively. Scale bar, 200 μm. (**B**) Kaplan–Meier survival analysis of neutrophil infiltration in PDAC, as defined by MPO expression. The p-value was obtained through the log-rank test. (**C**) Correlation between the extent of neutrophil infiltration in the tumor and the neutrophil-to-lymphocyte ratio in the peripheral blood of 60 patients with PDAC. (**D**) KPC-2 cells were transplanted into the pancreas of C57BL/6 mice to establish an orthotopic tumor model. Anti-Ly6G antibody was injected intraperitoneally in the orthotopic tumor mice at 1 week after transplantation to deplete neutrophils. Pancreatic tumors were harvested after 2 weeks of administration of Anti-Ly6G or IgG antibody. Representative in situ images and quantified graph of tumor weight. (**E**) Relative expression levels of N2 marker genes in HL60 cell-differentiated neutrophils and TANs. Expression levels of the *18 S* gene were used as an internal control (*n* = 3 per group). (**F**) Effects of TANs on the migration and invasion of pancreatic cancer cells (SUIT-2 and MIA PACA-2), as assessed using transwell assays (*n* = 3 per group). (**G**) Representative microphotographs of migrating (top) and invading (bottom) SUIT-2 cells. (**H**) Representative human cytokine array images (upper panels) of the supernatant derived from HL60-differentiated neutrophils and neutrophils treated with a conditioned medium of SUIT-2 cells. Red, blue, green, and black boxes represent CCL5, IGBP-1, CCL1, and positive control, respectively. The lower panels display the cytokine array images of supernatant from neutrophils isolated from mouse peripheral blood and TANs isolated from orthotopically implanted pancreatic tumors in mice. Red, blue, green, and black boxes represent CCL5, MIP1-gamma, TNFRII, and the positive control, respectively. (**I**) The qRT-PCR validation of *CCL5* mRNA expression in TANs. Relative expression levels of *CCL5* in neutrophils and TANs were normalized based on the corresponding expression levels of *18 S*. (*n* = 3 per group). (**J**) Immunohistochemistry of CCL5 in mice samples with or without TANs in the TME. Scale bar, 50 μm. (**K**) Gene expression profiling interactive analysis of *CCL5* in normal (gray bars) and tumor (red bars) samples from pancreatic cancer data from The Cancer Genome Atlas (TCGA) and Genotype-Tissue Expression databases. (**L**) Pearson correlation analysis of the mRNA expression levels of neutrophil signature (*S100A9 S100A8 CSF3R*) and *CCL5* in patients with PADC; data were extracted from the TCGA database. *p < 0.05, **p < 0.01, ***p < 0.001

Immunohistochemical (IHC) analysis for myeloperoxidase (MPO) in the resected PDAC tissues revealed substantial neutrophil infiltration in TME, especially adjacent to the malignant cells, in most cases (Fig. 1A**)**. Elevated TAN infiltration correlated with reduced overall survival in PDAC patients who underwent pancreatectomy (Fig. 1B). Additionally, a higher preoperative neutrophil-to-lymphocyte ratio (NLR), a predictor of poor prognosis in most solid tumors [21], positively correlated with increased TAN numbers in PDAC serum samples (Fig. 1C). This finding suggests that an increase in circulating neutrophils in the PB leads to high TAN infiltration. Furthermore, neutrophil depletion through anti-Ly6G antibody administration reduced TANs infiltration in a mouse model (online supplemental Fig. 1A–D) and slowed orthotopic pancreatic tumor growth (Fig. 1D and online supplemental Fig. 1E). These results implicate TANs in PDAC progression and poor prognosis.

### Polarization of neutrophils through pancreatic cancer cells

Next, we generated natural human neutrophils by differentiating HL60 cells, a human leukemia cell line, to investigate the detailed functions of TANs. Neutrophil’s characteristics were confirmed by segmented nuclear morphology and CD11b, CD14, and MPO expression status (online supplemental Fig. 2A–D). We induced TANs by treating neutrophils with conditioned medium from pancreatic cancer cell lines, SUIT-2 or MIAPACA-2. TANs exhibited upregulated expression of N2 marker genes, including *CD206*, *IL6*, *VEGF*, *MMP9*, *CXCR2*, and *TGFb* (Fig. 1E), and downregulated expression of N1 marker genes, including *TNFa*, *IL12a*, and *CXCL10* (online supplemental Fig. 2E). Moreover, the TAN-conditioned medium promoted the migration and invasion of SUIT-2 and MIAPACA-2, whereas that of original neutrophils did not (Fig. 1F and G and online supplemental Fig. 2F). These findings suggest that TANs’ protumor activity occurs in a paracrine manner, prompting us to investigate TAN-secreted molecules.

The cytokine antibody array revealed increased levels of several soluble factors, including CCL5, IGFBP-1, and CCL1, in the supernatants of TANs polarized from HL60-differentiated neutrophils (Fig. 1H). Neutrophils isolated from orthotopically implanted pancreatic tumors in mice (mouse TANs) abundantly secreted CCL5, MIP1-gamma, and TNFRII compared to circulating PB neutrophils (Fig. 1H). Moreover, CCL5 secretion was upregulated in both human and mouse TANs, highlighting its significance in these neutrophils. Upregulation of *CCL5* mRNA in TANs polarized from HL60 cells was confirmed by qRT-PCR (Fig. 1I). Conversely, CCL5 expression levels in the stroma were decreased in orthotopic pancreatic tumors of neutrophil-depleted mice (Fig. 1J), confirming TANs as a major source of CCL5 in the PDAC TME.

The Cancer Genome Atlas (TCGA) and Genotype-Tissue Expression project databases confirmed the elevated expression of *CCL5* mRNA in PDAC compared to normal pancreas (Fig. 1K). The Human Protein ATLAS (https://www.proteinatlas.org/) showed high CCL5 expression correlated with poor prognosis in PDAC patients (online supplementary Fig. 3A). Additionally, gene profiling using the gene expression profiling interactive analysis (GEPIA) website [15, 22] revealed a positive correlation between the neutrophil signature [15] and *CCL5* expression in PDAC (Fig. 1L). These findings indicate that pancreatic cancer cells stimulate TANs to secrete CCL5 into the microenvironment, potentially influencing the biological behavior of PDAC.

### TANs promote tumor progression through the CCL5-CCR5 axis

**Fig. 2:**
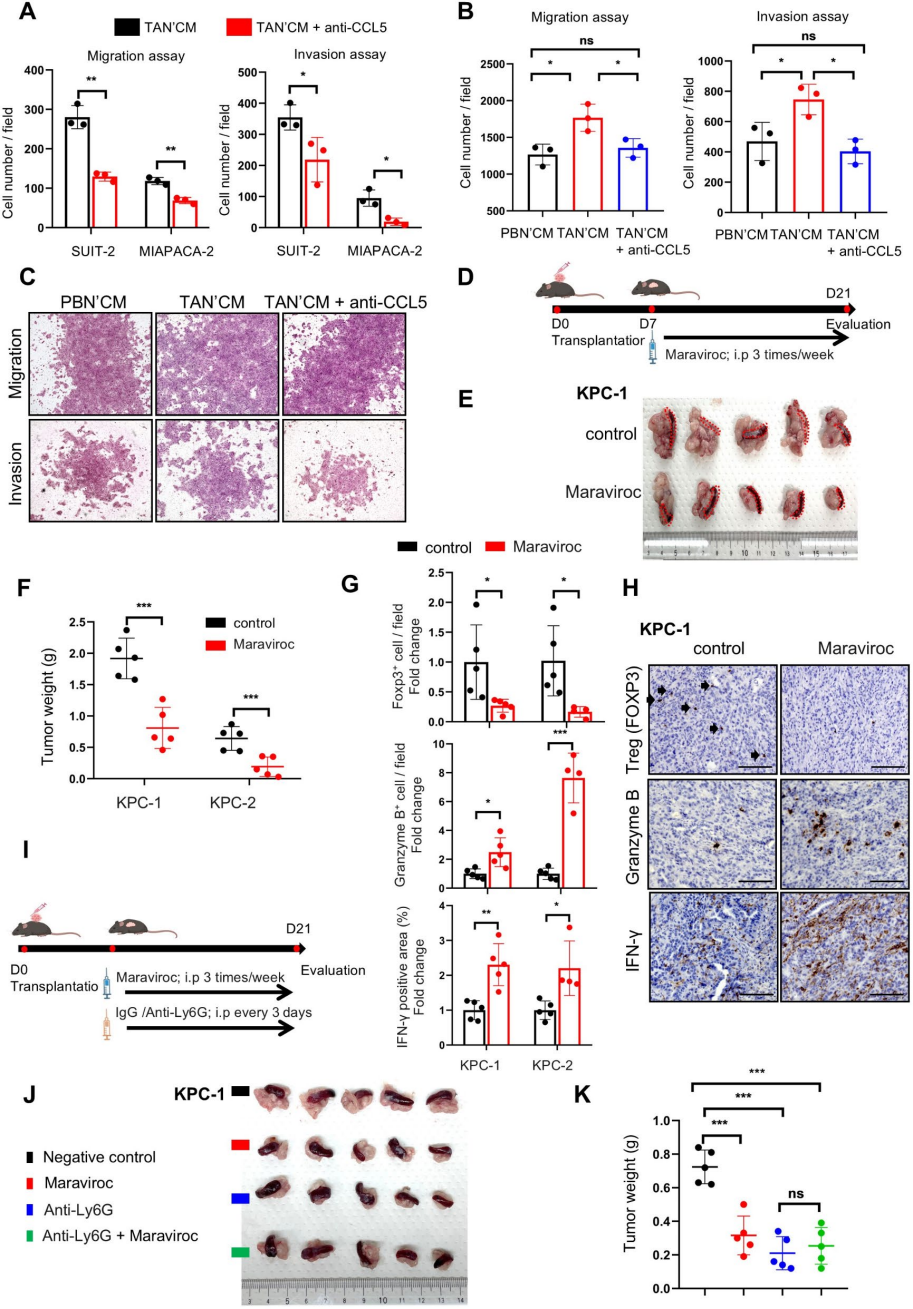
TANs promote tumor progression through the CCL5-CCR5 axis. (**A**) The anti-CCL5 neutralizing antibody suppressed the migration and invasion of SUIT-2 and MIA PACA-2 cells treated with conditioned TANs (TAN’CM) medium, as assessed using transwell assays (*n* = 3 per group). (**B**) Migration and invasion of KPC-1 mouse pancreatic cancer cells treated with conditioned medium from neutrophils in mouse peripheral blood (PBN’CM) or those isolated from orthotopic pancreatic tumors (TAN’CM) with or without the anti-CCL5 antibody. (**C**) Representative microphotographs of migrating (top) and invading (bottom) KPC-1 cells. (**D**) Experimental schema demonstrating the orthotopic implantation of KPC-1 or KPC-2 cells into C57/B6 mice, *n* = 5. (**E**) Representative in situ images of tumors from KPC-1 cells and (**F**) quantified graphs of tumor weight in KPC-1 or KPC-2-transplanted mice after treatment. (**G**) Quantification for the immunohistochemistry of FOXP3^+^, Granzyme B^+^, and IFN-γ^+^ cells in orthotopically transplanted mice tumors. (**H**) Representative images for the immunohistochemistry of FOXP3^+^, Granzyme B^+^, and IFN γ^+^ cells in orthotopically transplanted mouse tumors. Arrows indicate positive staining. Scale bar, 100 μm. (**I**) Experimental schema showing the orthotopic implantation of KPC-2 cells into female C57/B6 mice and subsequent intraperitoneal injection with anti-Ly6G and maraviroc; *n* = 5. (**J**) Photographs of resected tumors and (**K**) quantified graphs of tumor weight in KPC-2-transplanted mice after treatment. *p < 0.05, **p < 0.01, ***p < 0.001; ns, not significant

To further validate the effect of CCL5 secreted from TANs, we employed a neutralizing antibody to inhibit its activity. Treatment with an anti-CCL5 antibody in the conditioned medium of TANs led to suppressed migration and invasion of SUIT-2 and MIAPACA-2 cells (Fig. 2A and online supplemental Fig. 3B). Similarly, the supernatant of neutrophils isolated from orthotopic pancreatic tumors in mice promoted the migration and invasion of KPC-1 cells compared to that from PB neutrophils. However, this effect was abrogated upon CCL5 neutralization (Fig. 2B–C). Treatment with anti-CCL5 antibody in SUIT-2 and MIAPACA-2 cells without conditioned medium from TANs did not change their migratory and invasive ability (online supplemental Fig. 3C-D).

Given the critical role of CCL5 in cancer cell activity [23] and its high affinity for the receptor CCR5, we investigated whether targeting the CCL5–CCR5 axis could be an effective strategy in PDAC treatment using maraviroc, a selective CCR5 antagonist. Repeated administration of maraviroc to orthotopically transplanted mice (Fig. 2D) resulted in suppressed pancreatic tumor progression (Fig. 2E–F and online supplemental Fig. 3E–F). Additionally, maraviroc treatment led to reduced cancer cell dissemination and ascites production in transplanted mice (online supplemental Fig. 3G). Since CCL5 is known to recruit Treg cells into the TME [24], we examined the immune status of maraviroc-treated pancreatic tumors. Further, IHC analysis revealed that maraviroc attenuated Treg cell infiltration into the TME, which was accompanied by a marked increase in the expression of the cytotoxic factors Granzyme B and IFN-γ in the tumor area (Fig. 2G–H and online supplemental Fig. 3H). To further determine the role of CCL5 in TANs, mice were treated with anti-Ly6G to deplete neutrophils, and then maraviroc was administered peritoneally (Fig. 2I). Maraviroc administration in neutrophil-depleted mice did not suppress tumor growth, indicating that CCL5-CCR5 signaling induced by TANs is a key factor in tumor progression. (Fig. 2J-K and online supplemental Fig. 3I).

### TANs regulate protumor immunity through the interaction with CD8 + T cells

**Fig. 3:**
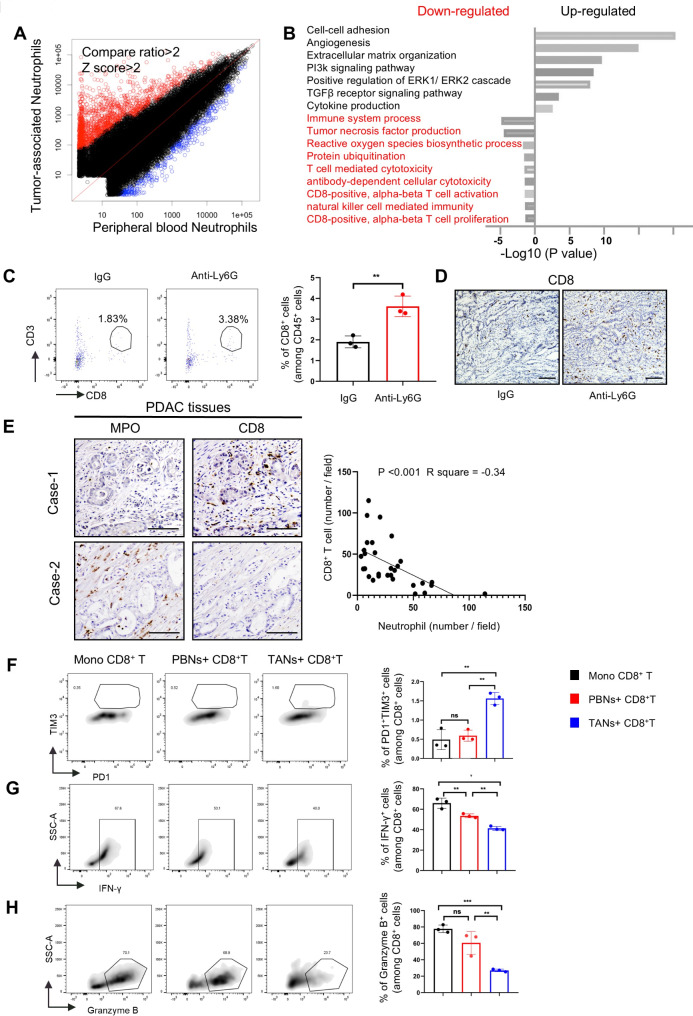
TANs suppress CD8 + T cells infiltration and their antitumor activities. (**A**) A gene plot for microarray analysis between TANs and peripheral blood neutrophils from orthotopically transplanted mouse tumors. (**B**) Top-ranked enriched pathways that were upregulated (black) and downregulated (red) in TANs compared with those in mouse peripheral blood neutrophils, as determined using gene ontology analysis. (**C**) Representative images of flow cytometry and (**D**) immunohistochemistry for CD8^+^ T-cells in orthotopically transplanted mouse tumors with or without neutrophil depletion. CD45^+^ cells are presented in the flow cytometry. Scale bars, 100 μm. (**E**) Representative images of immunohistochemistry for neutrophils (MPO) and CD8^+^ cells in the resected PDAC tissues. Scale bars, 100 μm (left). Case-1 represents severe TAN infiltration with few CD8 cells, whereas Case-2 represents limited TAN infiltration with abundant CD8 cells. The correlation between the number of neutrophils and CD8^+^ cells per field of view at 200× magnification in PDAC tissues was statistically analyzed (right, *n* = 31). (F–H) The percentages of PD1^+^TIM3^+^CD8^+^ T- (**F**), IFN-*γ*-producing CD8^+^ T- (**G**), and Granzyme B-producing CD8^+^ T- (**H**) cells were evaluated using flow cytometry (*n* = 3). **p < 0.01, ***p < 0.001; ns, not significant

Next, we performed microarray analysis for neutrophils isolated from pancreatic tumors and PB in orthotopically transplanted mice to assess transcriptomic alterations associated with their protumor activity (Fig. 3A). Gene ontology analysis revealed downregulation of pathways associated with antitumor immunity, such as ROS generation, T-cell, and NK cell-mediated immunities, in TANs. Conversely, enrichment of pathways related to angiogenesis and TGFβ receptor signaling, contributing to tumor growth, was observed (Fig. 3B). Several pathways related to CD8^+^ T-cell activation were downregulated in TANs, and further analysis of previous neutrophil depletion experiments in the orthotopic mouse model demonstrated that infiltration of CD8^+^ T-cell in the TME markedly increased in the absence of TANs (Fig. 3C–D). Additionally, a negative correlation between TANs and CD8^+^ T-cell infiltration was observed in human PDAC tissues (Fig. 3E).

We investigated the direct effect of TANs on the antitumor activity of CD8^+^ T-cells by co-culturing neutrophils isolated from mice with autologous CD8^+^ T-cells, which were purified from the spleen and activated with anti-CD3/CD28 beads. Neutrophils isolated from orthotopic pancreatic tumors accelerated CD8^+^ T-cell dysfunction, defined as the increased percentage of PD1^+^TIM3^+^ cells, and inhibited Granzyme B and IFN-γ production, whereas PB neutrophils did not (Fig. 3F–H and online supplemental Fig. 8).

### Nectin2 expression is upregulated in TANs and related to protumor immunity

**Fig. 4:**
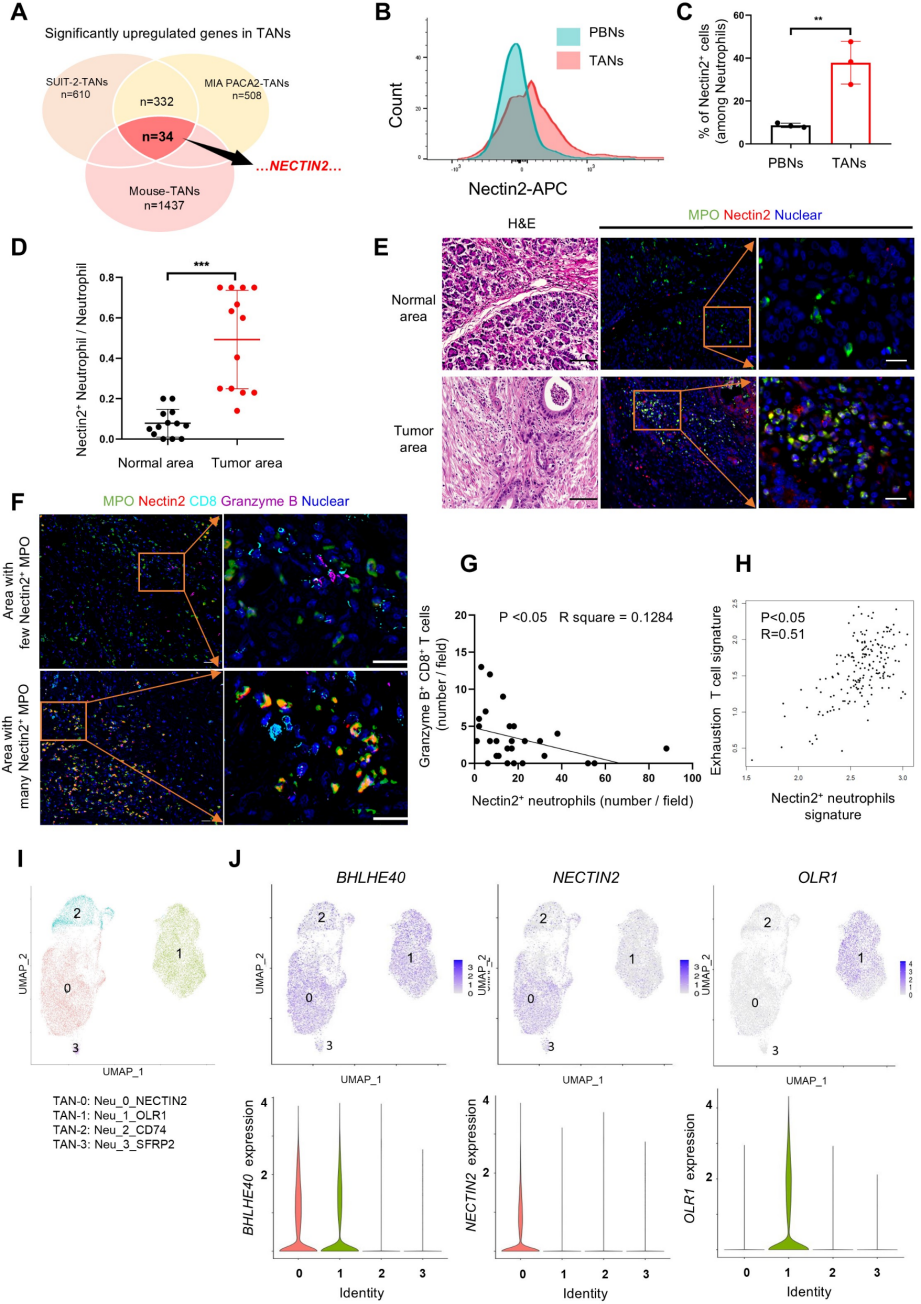
Nectin2 expression increased in tumor-associated neutrophils and was related to protumor immunity. (**A**) Microarray analysis results show the number of genes whose expression in TANs was upregulated under varying experimental conditions. (**B**) and (**C**) Flow cytometry analysis of Nectin2 expression in neutrophils from peripheral blood (PBNs) and tumor-associated neutrophils (TANs) in mice. The percentage of Nectin2^+^ neutrophils is shown. (**D**) and (**E**) indicate the percentage of Nectin2^+^ neutrophils (double positive for Nectin2 and MPO) of all neutrophils in the tumor area and adjacent normal pancreas in resected PDAC tissues (D, *n* = 13). Representative images of H&E and immunofluorescence for MPO (green) and Nectin2 (red) in the adjacent normal pancreas (top panels) and tumor area (bottom panels) (**E**). Yellow indicates Nectin2^+^ TANs. Scale bar, 100 μm in H&E and 20 μm in immunofluorescence. (**F**) Representative images of multi-immunohistochemistry for Nectin2^+^ neutrophils (double positive for Nectin2 and MPO) and Granzyme B^+^ CD8^+^ T-cells (double positive for Granzyme B and CD8) in PDAC. Case-1 shows an area with less Nectin2^+^ TAN infiltration and abundant Granzyme B^+^ CD8^+^ T-cells. Case 2 shows an area with severe Nectin2^+^ TANs infiltration and fewer Granzyme B^+^ CD8^+^ T-cells. Scale bars, 20 μm. (**G**) The number of Nectin2^+^ neutrophils and Granzyme B^+^ CD8^+^ cells per field of view at 200× magnification, as counted in 35 fields from seven PDAC tissues. (**H**) Pearson correlation analysis of the mRNA levels in Nectin2^+^ neutrophil (*CD112*,* S100A9*,* S100A8*,* CSF3R*) and exhausted T-cell (*HAVCR2*,* TIGIT*,* LAG3*,* PDCD1*,* CXCL13*,* LAYN*) signatures in 177 human PDAC from the TCGA database. (**I**) Single-cell RNA sequence analysis of the public dataset GSE205013 [26], which comprises cells from 27 freshly collected human PDAC samples. After segregation of neutrophils from CD45^+^ cells in the tumors as shown in online supplemental Fig. 6A–B, uniform manifold approximation and projection (UMAP) visualization re-clustered neutrophils into 4 clusters (0: TAN-0_NECTIN2, 1: TAN-1_OLR1, 2: TAN-2_CD74, 3: TAN-3_SFRP2). Detailed characteristics of each cluster are shown in the online supplemental Table 1. (**J**) UMAP of neutrophils separately featuring the expression of *BHLHE40* (left), *NECTIN2* (middle), and *OLR1* (right) in each population. **p < 0.01, ***p < 0.001

We further investigated how TANs suppressed antitumor immunity by CD8^+^ T-cells. Microarray analysis detected 1437 and 332 upregulated genes in mouse-derived TANs and human neutrophils treated with conditioned SUIT-2 and MIA PACA-2 media, respectively. Among these, 34 genes were upregulated in both human and mouse TANs (Fig. 4A and online supplemental Fig. 4A–B), including *Nectin2*, which modulates T-cell function as an immune checkpoint molecule [25]. 

Based on these findings, we hypothesized that increased Nectin2 expression in TANs inhibits CD8^+^ T-cell function. To test this hypothesis, we validated the increased mRNA and protein expression of *Nectin2* in human- and mouse-derived TANs using qRT-PCR and western blotting (online supplemental Fig. 4C–4 F). Cell surface expression of Nectin2 was also heightened in mouse-isolated TANs (Fig. 4B–C). Immunofluorescence revealed that neutrophils expressing Nectin2 (Nectin2^+^ neutrophils) were more frequently observed in the TME of PDAC than in the adjacent normal pancreatic tissues (Fig. 4D–E**).** Additionally, TCGA analysis revealed considerably elevated *Nectin2* expression in pancreatic tumors compared to normal pancreatic tissues (online supplemental Fig. 4G).

Multiple IHC staining identified a few Granzyme B^+^ CD8^+^ T-cells in areas with elevated Nectin2^+^ neutrophil contents (Fig. 4F). The number of Granzyme B^+^ CD8^+^ T-cells in the TME showed a negative correlation with that of Nectin2^+^ neutrophils (Fig. 4G**).** Moreover, the GEPIA revealed a positive correlation between Nectin2^+^ neutrophils in PDAC and exhausted T-cell signatures (Fig. 4H). Nectin2 expression on pancreatic cancer cells showed no relationship with the status of T cell exhaustion. (online supplemental Fig. 4H).

The single-cell transcriptomic profile of 27 patients with PDAC in the GSE20501 dataset from the Gene Expression Omnibus database (https://www.ncbi.nlm.nih.gov/geo/) was analyzed to validate Nectin2 expression in TANs within human PDAC [26]. Four TAN clusters, TAN-0, TAN-1, TAN-2, and TAN-3, were identified based on specific neutrophil signatures in the CD45^+^ cluster of the dataset (Fig. 4I, online supplemental Fig. 5A–B and online supplemental Table 1). Of these, TAN-0 and TAN-1 exhibited high expression of *BHLHE40*, indicating their role as tumor-promoting TANs. *NECTIN2* was exclusively expressed in TAN-0, suggesting that Nectin2^+^ neutrophils are a subpopulation of tumor-promoting TANs (Fig. 4J). In contrast, TAN 1 exclusively expressed *OLR1*, a marker of immunosuppressive neutrophils in other solid tumors (Fig. 4J) [27]. These results indicate that TANs with *BHLHE40* expression comprise the Nectin2^+^ and OLR1^+^ phenotypes, which might promote tumor growth through different mechanisms. Nectin2^+^ expression in the neutrophils was increased in the TME and may establish an immunosuppressive microenvironment in PDAC by suppressing CD8^+^ T-cell activity.

### Inhibition of Nectin2 retrieves antitumor ability in CD8 + T cells

**Fig. 5:**
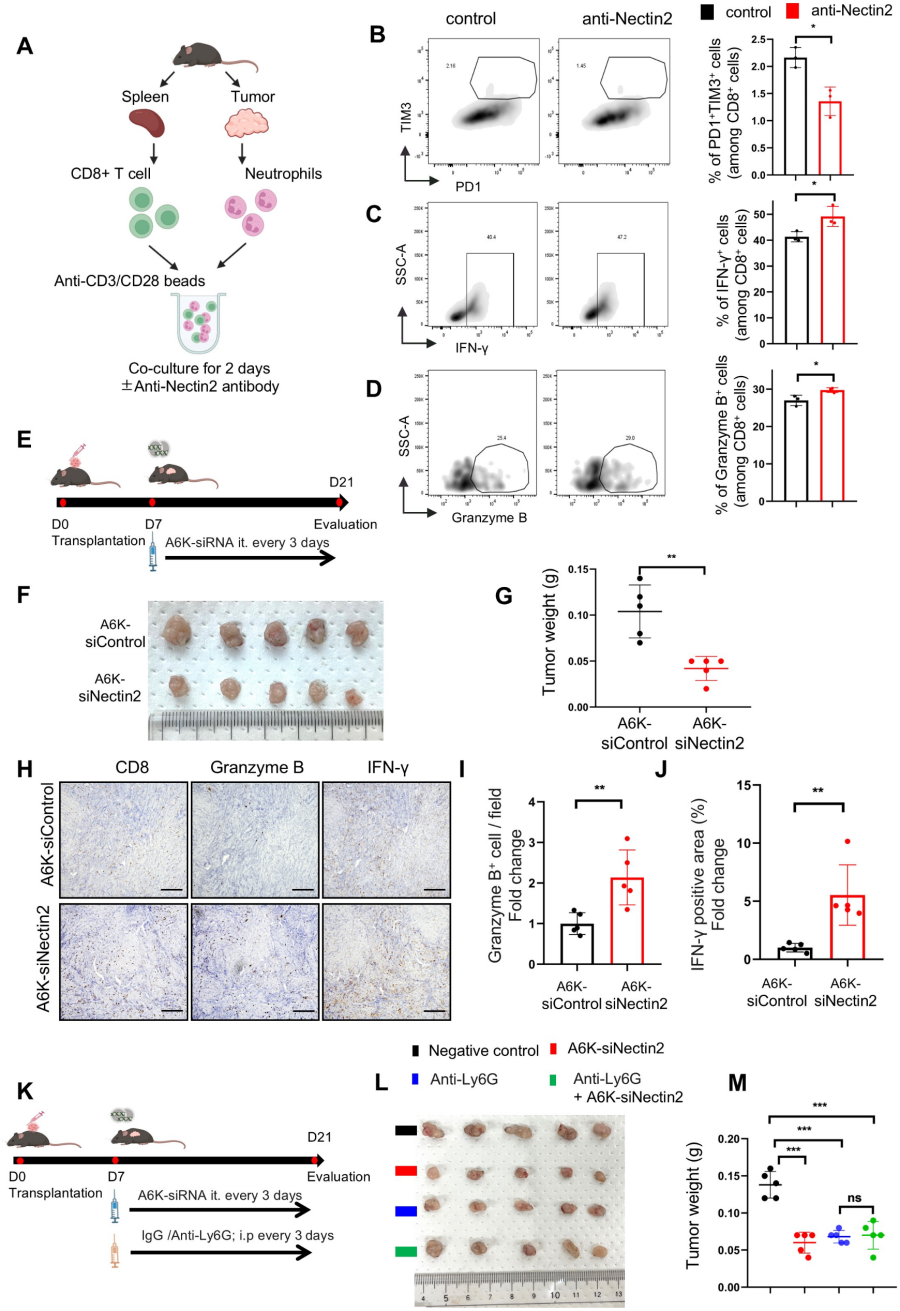
Inhibiting Nectin2 increases antitumor ability in CD8 + T cells. (**A**) Experimental schema showing the co-culture of CD8^+^ T-cells with autologous neutrophils from KPC tumors with or without anti-Nectin2 antibody. (**B**-**D**) The percentages of PD1^+^TIM3^+^CD8^+^ T- (B), IFN-γ-producing CD8^+^ T- (**C**), and granzyme B-producing CD8^+^ T- (**D**) cells calculated using flow cytometry (*n* = 3). (**E**) Experimental schema showing the subcutaneous implantation of KPC-2 cells into female C57/B6 mice and subsequent intratumoral injection with A6K-siNectin2 or A6K-siRNA control (A6K-siControl); *n* = 5. (**F**) Photographs of resected tumors treated with A6K-siControl or A6K-siNectin2. (**G**) Graphs showing tumor weight after A6K-siNectin2 treatment. (**H**) Representative immunohistochemistry of CD8, Granzyme B, and IFN-γ in the serial sections of the subcutaneous tumors triggered through transplantation of KPC-2 cells. Scale bar, 200 μm. (**I** and **J**) Statistical analysis of Granzyme B^+^ (**I**) and IFN γ^+^ (**J**) cells in the tumors; *n* = 5. (**K**) Experimental schema showing the subcutaneous implantation of KPC-2 cells into female C57/B6 mice and subsequent intraperitoneal injection with anti-Ly6G and intratumoral injection with A6K-siNectin2; *n* = 5. (**L**) Photographs of resected tumor and (**M**) quantified graphs of tumor weight in KPC-2-transplanted mice after treatment. *p < 0.05, **p < 0.01, ***p < 0.001

To investigate the direct effect of Nectin2^+^ neutrophils on the CD8^+^ T-cells, we blocked Nectin2 in TANs with a neutralizing antibody in the co-culture system of TANs and CD8^+^ T-cells (Fig. 5A). The blockade of Nectin2 in TANs reduced the percentage of the PD1^+^TIM3^+^ cells and increased that of the cytotoxic markers Granzyme B and IFN-γ in co-cultured CD8^+^ T-cells (Fig. 5B–D). To inhibit Nectin2 in the TME in *vivo* experiment, we used a complex of A6K and siRNA for Nectin2 (A6K-siNectin2), which revealed a sufficient knockdown effect in vitro (online supplemental Fig. 6A–B). Intratumoral injection of A6K-siNectin2 effectively suppressed the membranous expression of Nectin2 in TANs (Fig. 5E and online supplemental Fig. 6C–D). Moreover, the weight and volume of tumors treated with A6K-siNectin2 were lower than those treated with A6K-control siRNA (Fig. 5F–G and online supplemental Fig. 6E). CD8^+^ T-cells in the tumors of *Nectin2* knockdown mice upregulated Granzyme B and IFN-γ expression (Fig. 5H–J). To further confirm whether this tumor alleviation effect upon Nectin2 knockdown was brought about by neutrophils, we administrated A6K-siNectin2 in the neutrophil-depleted mice. Neutrophil depletion reduced tumor weight and volume, while the injection of A6K-siNectin2 did not further inhibit tumor progression. (Fig. 5K–M and online supplemental Fig. 6F). These findings indicate that membranous Nectin2 expression in TANs mediates the immunosuppressive TME by suppressing CD8^+^ T-cell antitumor immunity.

### Endoplasmic reticulum (ER) stress increased in TANs owing to their protumor function

**Fig. 6:**
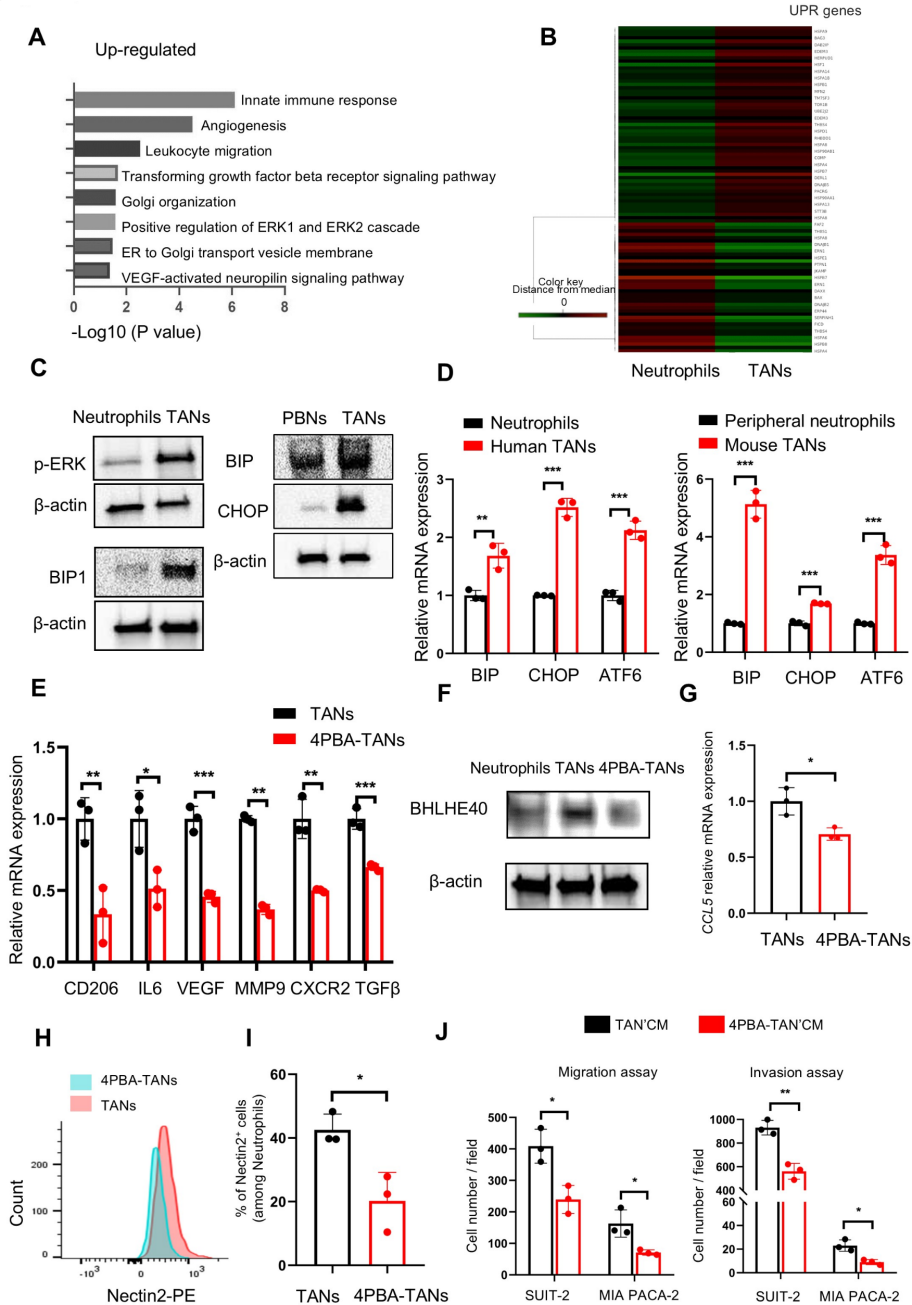
ER stress increased in TANs, producing protumor functions. (**A**). Gene ontology analysis of genes whose expression was upregulated in human TANs compared with original neutrophils. (**B**) Heatmap of genes involved in the response to unfolded proteins (UPR) between neutrophils and TANs. (**C**) Western blotting results show ER stress-related proteins (p-ERK and BIP1) in human neutrophils differentiated from HL60 cells and those treated with the supernatant of SUIT-2 cells (TANs) (left panels). Western blotting results identifying ER stress-related proteins (BIP and CHOP) in neutrophils from peripheral blood (PBNs) and orthotopically implanted tumors (TANs) in mice (right panels). β-actin was used as a loading control. (**D**) Relative expression levels of ER stress-related genes in humans (left panel, *18 S* served as an internal control) and mouse (right panel, GAPDH served as an internal control) neutrophils during phenotype switch. (*n* = 3 per group). (**E**) Relative expression levels of N2 marker genes in human (TANs) and 4-Phenylbutyric acid (4-PBA)-pretreated TANs (4PBA-TANs). The mRNA expression level of each gene was normalized to fold over *18 S*. (*n* = 3 per group). (**F**) Western blotting results demonstrating BHLHE40 expression in neutrophils, TANs, and 4PBA-TANs. β-actin was used as a loading control. (**G**) Relative expression levels of *CCL5* mRNA in TANs and 4PBA-TANs. (*n* = 3 per group). (**H**-**I**) Flow cytometry analysis of Nectin2 expression in TANs and 4PBA-TANs (**H**). The percentage of Nectin2 + neutrophils (**I**). (**J**) The migration and invasion of SUIT-2 and MIA PACA-2 cells treated with TAN’CM or 4PBA-TAN’CM, as assessed using transwell assays (*n* = 3 per group). *p < 0.05, **p < 0.01, ***p < 0.001

Finally, we sought to elucidate the critical factor of modulating protumor function in TANs as a candidate for treating PDAC. Microarray data revealed enriched ER and Golgi organization-related signaling pathways in human TANs (Fig. 6A). ER stress significantly affects the functions of myeloid cells [27]; therefore, we focused on ER stress as an inducer of protumor activities in neutrophils. Heatmap revealed that activities associated with response to unfolded protein were upregulated in HL-60 derived TANs (Fig. 6B) and those isolated from orthotopically transplanted tumors in mice (online supplemental Fig. 7A). The ER-related pathway and proteins, p-ERK and BIP, were upregulated in human and mouse TANs compared with neutrophils (Fig. 6C). The expression levels of genes downstream of ER stress, namely *BIP*,* CHOP*, and *ATF6*, were elevated in TANs, confirming that ER stress signaling is activated during the phenotype switch of neutrophils (Fig. 6D).

To further explore whether ER stress modulates tumor-promoting characters in TANs, we treated neutrophils with 4-phenylbutyric acid (4-PBA), an ER stress inhibitor, before polarizing them with a conditioned medium from pancreatic cancer cells. Our findings revealed downregulation of N2 marker genes in TANs pretreated with 4-PBA, indicating that the protumor function of TANs was diminished in the absence of ER stress activation (Fig. 6E). Similarly, 4-PBA prevented the upregulation of BHLHE40 in TANs, indicating that ER stress activation may trigger the phenotype switch in neutrophils (Fig. 6F). On the other hand, N1 marker expression in TANs did not change under treatment with ER inhibitor (online supplemental Fig. 7B). Pretreatment of TANs with 4-PBA decreased *CCL5* expression and surface Nectin2 expression (Fig. 6G-I). Furthermore, the functional impact of TANs on cancer cell migration and invasion was also inhibited by ER stress inhibition (Fig. 6J and online supplemental Fig. 7C-D). Autophagy is a cellular protective mechanism for ER stress and is activated to remove damaged or aggregated cellular proteins in the ER [28, 29]. Treatment of neutrophils with conditioned medium from pancreatic cancer cells upregulated the expression of LC3-II (online supplemental Fig. 7E-G). These findings indicate that ER stress occurs via signals from pancreatic cancer cells in neutrophils and plays a key role in the protumor function of TANs.

## Discussion

**Fig. 7:**
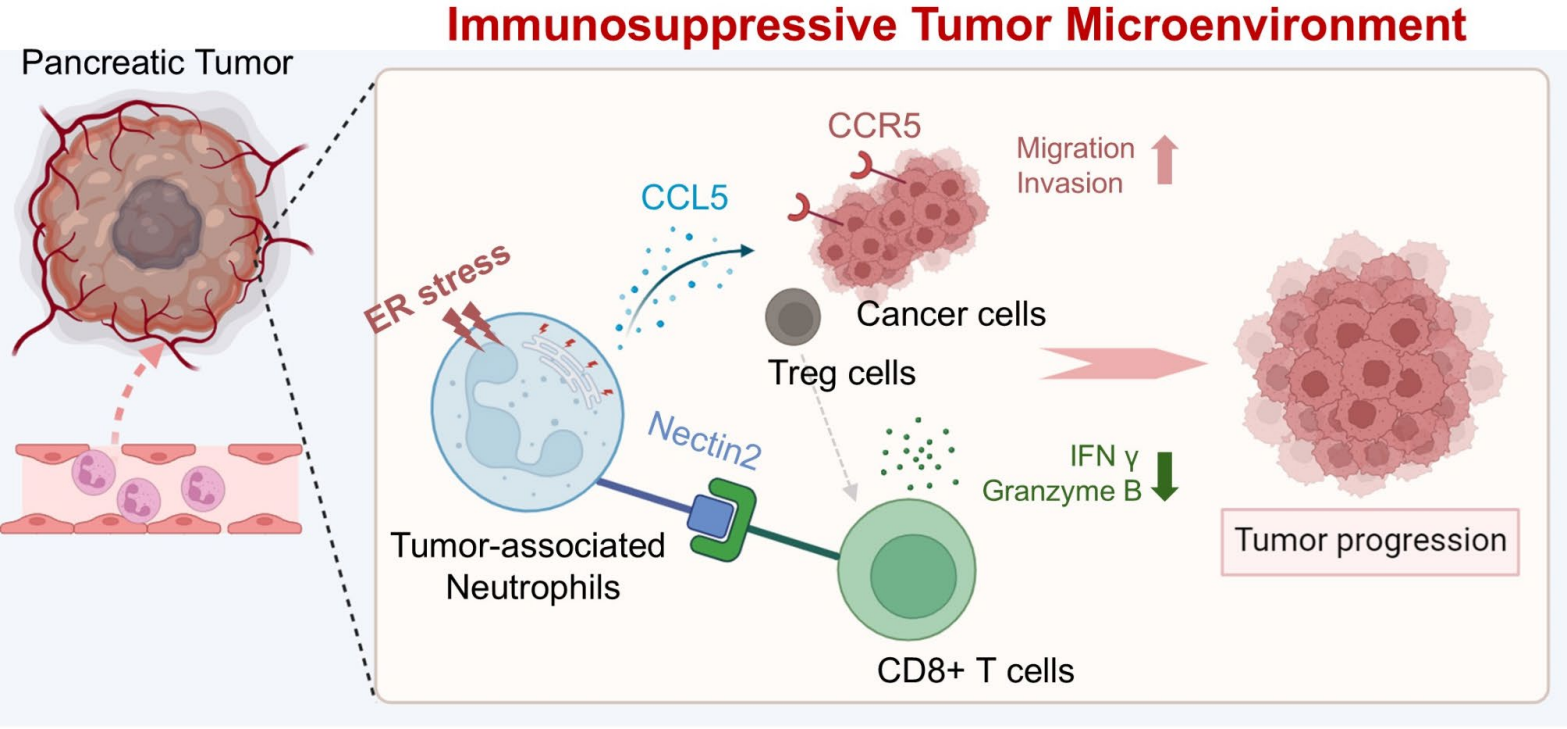
Graphical summary depicting the role of tumor-associated neutrophils in the immunosuppressive tumor microenvironment of PDAC. Neutrophils stimulated by cancer cells become the protumor phenotype through increased ER stress. Polarized tumor-associated neutrophils upregulate CCL5 secretion, which promotes cancer cell migration and invasion and enhances Treg cell infiltration in the tumor. In addition, a subpopulation of tumor-associated neutrophils upregulates Nectin2 expression, directly impeding the secretion of IFN-γ and Granzyme B by CD8^+^ T-cells through immune checkpoint signaling. Blockade of the CCL5–CCR5 axis or Nectin2 boosted CD8^+^ T-cell cytotoxicity, inhibiting tumor progression in PDAC

This study elucidated the molecular mechanisms underlying the protumor activity of TANs by comprehensively analyzing various neutrophils originating from human and mouse specimens (Fig. 7). Through cytokine array and microarray analyses, novel key molecules of TANs, such as CCL5 and Nectin2, were identified. These molecules contribute to the immunosuppressive TME in PDAC and result in tumor progression. CCL5, abundantly secreted in TANs, promotes the migration and invasion of pancreatic cancer cells and facilitates the recruitment of Treg cells. Nectin2, expressed in a subpopulation of TANs, induces CD8^+^ T-cell dysfunction. Blockade of the CCL5–CCR5 axis or Nectin2 improved CD8^+^ T-cell activity and suppressed tumor growth in vivo. Overall, this study highlights the roles of CCL5 and Nectin2 mediating the protumor activity of TANs, particularly in establishing an immunosuppressive TME in PDAC.

The emergence of immune checkpoints has revolutionized cancer therapy. However, immune checkpoint blockade therapies targeting PD-1 or CTLA-4 show limited efficacy against PDAC [30, 31], highlighting the urgency of finding new effective targets. Nectin2, a cell adhesion molecule and immune checkpoint ligand [32], inhibits the cytotoxic activities of CD8^+^ T- and NK-cells by interacting with CD112R and TIGIT [33–35]. However, its significance in PDAC or its expression in TANs remained unexplored. In this study, we identified *Nectin2* as one of the genes whose expression was consistently upregulated in both human- and mouse-derived TANs. Analyses of resected PDAC specimens, public data, and single-cell transcriptomes from PDAC patients confirmed the heightened expression of *Nectin2* in TANs, correlating with immunosuppression in PDAC. Furthermore, we showed that Nectin2^+^ TANs suppress the antitumor activity of CD8^+^ T-cells, affirming the direct suppressive effect of Nectin2 on cytotoxic T-cells within TANs. While previous studies have suggested the paracrine nature of neutrophils’ suppressive effects on T-cell functions, such as arginase 1 and NO secretions, our findings demonstrate that TANs upregulate Nectin2 expression, creating an immunosuppressive microenvironment through direct interaction with CD8^+^ T-cells [12, 36]. Together, these results suggest that Nectin2 could be a unique and promising therapeutic target against refractory PDAC, facilitating the development of novel and effective immunotherapies.

A novel protumor mechanism of TANs, wherein polarized neutrophils create an immunosuppressive TME through CCL5 and Nectin2 expression under the influence of the TME, has been identified. Neutrophils are plastic and exhibit functional diversity through maturation, aging, and tissue-specific signals [37]. They have dual effects on tumor progression and anti- and protumor activity, which are exerted by changing their functional phenotypes according to circumstances. However, the heterogeneity of neutrophils has been less extensively studied [11] due to their experimental difficulties, vulnerable nature, and short half-life in vitro [38, 39]. LOX1 (*OLR1*-encoded) is a differentially expressed immunosuppressive phenotype found in low-density neutrophils, and increased tumor infiltration of LOX1^+^ neutrophils is associated with poor clinical outcomes in colon and lung cancers [27]. LOX1^+^ TANs highly upregulate the expression of nitric oxide, ROS, and arginase 1 and suppress T-cell proliferation. In pancreatic cancer, exogenous LOX1 expression has been reported to promote epithelial-mesenchymal transition in cancer cells and is correlated with poor prognosis [40]. In this study, transcriptomic data from patients with PDAC revealed that Nectin2^+^ TANs constitute a subpopulation of BHLHE40^+^ TANs distinct from LOX1^+^ TANs. Nectin2^+^ TANs inhibit CD8^+^ T-cell activity by inducing T-cell dysfunction. Given that LOX1^+^ TANs suppress T-cell proliferation by secreting ROS and arginase 1, as previously reported [27], it suggests that there is functional diversity among the immunosuppressive phenotypes of TANs, which modulate antitumor immunity through different mechanisms. Furthermore, microarray analysis identified diminished pathway enrichment associated with NK cell activity in TANs. As the Nectin2 receptor is also expressed in NK cells, the interaction between Nectin2^+^ TANs and NK cells warrants further exploration.

CCL5 is crucial for immune cell recruitment to the sites of inflammation during an immune response. In cancer, tumor and stromal cells secrete CCL5, which promotes tumor progression and chemoresistance by interacting with its receptor, CCR5, found on various immune cells [23, 41]. CCL5–CCR5 signaling facilitates the immunosuppressive polarization of monocytes and myeloid cells, contributing to an immunosuppressive TME in various cancers, including breast cancer, colorectal cancer, and Hodgkin lymphoma [42–44]. In PDAC, tumor cells express CCL5 and CCR5, and CCL5–CCR5 signaling promotes cancer cell proliferation, invasion, and metastasis [45, 46]. While several CCR5 antagonists inhibiting this axis are under clinical investigation [23], the contribution of neutrophils to this axis remains unknown.

In this study, our comprehensive analyses demonstrate that TANs contribute to CCL5–CCR5 signaling in PDAC and secrete abundant CCL5 into the TME, enhancing cancer cell migration, invasion, and Treg cell infiltration. Furthermore, maraviroc, a CCR5 antagonist, restored antitumor immunity in orthotopically transplanted pancreatic tumors in mice, indicating that targeting the CCL5–CCR5 axis is a promising immunotherapy for PDAC. Clinical trials to determine the efficacy of combined treatment with a dual antagonist of CCR2/CCR5 (BMS-813160) and anti-PD-1 nivolumab with or without granulocyte-macrophage colony-stimulating factor-secreting allogeneic PDAC vaccine against locally advanced PDAC are ongoing (NCT03767582). However, further studies are required to clarify the specific role of neutrophil-derived in the immunosuppressive TME of PDAC.

As polarized TANs exert protumor activities in PDAC, preventing phenotypic changes may inhibit tumor progression. Our study shows that ER stress triggered by cancer cells induces protumor activity in TANs. This finding aligns with previous studies showing the upregulation of ER stress-related genes in the immunosuppressive phenotype of neutrophils isolated from the spleens of KPC mice or PB of patients with non-small cell lung cancer and head and neck cancers [27, 47]. Chemicals inducing ER stress, such as thapsigargin or dithiothreitol, could convert neutrophils isolated from the PB of healthy donors into an immunosuppressive phenotype characterized by LOX-1 expression in vitro [27]. Furthermore, ER stress in TANs induced the expression of BHLHE40, including *VEGFA*, *PLAU*, *LGALS3*, and *LDHA* [18]. Consistent with these findings, we confirmed that 4-PBA-inhibited ER stress abrogated the upregulation of N2 marker genes in TANs, which may indicate the involvement of ER stress in altering the TAN phenotype. On the other hand, N1 marker expression in TANs did not change under treatment with ER inhibitor, unlike N2 markers. This finding may be inconsistent with the notion that ER stress is a key regulator of phenotype switching. Previous studies have shown that the N1 markers TNFα, IL12, and CXCL10 are not only associated with neutrophils’ anti-tumor activity but also linked to ER stress signaling [48–50]. Therefore, inhibiting ER stress in TANs might lead to a direct response in these markers, potentially complicating the interpretation of phenotype switching. Further comprehensive investigation into the anti-tumor activities of TANs is necessary to reach a definitive conclusion.

Our study has several limitations. First, we performed in vitro functional experiments of TANs using HL-60 derived neutrophils instead of isolating neutrophils from PDAC or healthy patients. The results obtained from these differentiated neutrophils may not reflect the real status of neutrophils in the body, although the HL-60 cell line is a widely accepted model for human neutrophils. Therefore, we extensively verified the findings from HL-60 experiments using neutrophils isolated from PB and orthotopic PDAC tumors in mice. Expression of Nectin2 in TANs was also confirmed by immunofluorescence staining and single-cell RNA sequence analysis in human PDAC tissues. Second, the current study lacks specific inhibition to target Nectin2 + TANs. Administration of A6K-siNectin2 in vivo tumors may have suppressed the tumor growth by acting on not only Nectin2 + TANs but also cancer cells that express Nectin2. To address this issue, we investigated the effect of A6K-siNectin2 on tumor growth in a neutrophil-depleted mice model. Any effect of Nectin2 knockdown was not observed in the tumor without TANs, indicating that Nectin2 expressed in TANs is the one that exerts protumor activity. To further confirm the function of Nectin2 on TANs, analysis using neutrophil-specific conditional knockout mice will be required. Finally, our analysis of heterogeneity in TANs is not sufficient. We have identified two immunosuppressive phenotypes of TANs, LOX + TANs and Nectin2 + TANs. The function of Nectin2 + TANs has been elucidated deeply in this study, while the LOX + TANs were not subject to our analysis. It is unclear whether targeting only Nectin2 + TANs is sufficient to overcome the immunosuppressive microenvironment, or whether control of both Nectin2 + TANs and LOX + TANs is necessary. Functional differences in these two phenotypes and their interactive actions remain to be revealed.

## Conclusions

This study highlights the clinical significance of infiltrating neutrophils in PDAC and elucidates the mechanism underlying their protumor effects. Tumor-associated neutrophils are polarized by TME-triggered ER stress and promote PDAC progression by upregulating CCL5 and Nectin2 expression. Specifically, Nectin2^+^ TANs, a subpopulation of immunosuppressive TANs in PDAC, directly inhibit the cytotoxic activity of CD8^+^ T-cells by acting as immune checkpoint ligands. Targeting TAN-specific molecules or preventing their phenotype switching could be a novel strategy for improving immunotherapeutic approaches to treat PDAC.

## Electronic supplementary material

Below is the link to the electronic supplementary material.

Supplementary Material 1

Supplementary Material 2

Supplementary Material 3

Supplementary Material 4

Supplementary Material 5

Supplementary Material 6

Supplementary Material 7

Supplementary Material 8

Supplementary Material 9

Supplementary Material 10

Supplementary Material 11

## Data Availability

Microarray data have been deposited at GEO and will be publicly available as of the date of publication. The data generated in this study are available upon request from the corresponding author (ikenaga.naoki.533@m.kyushu-u.ac.jp).

## Abbreviations

ATF6: Activating Transcription Factor 6
BHLHE40: Basic Helix-Loop-Helix Family Member E40
BIP: Binding Immunoglobulin Protein
CCL5: Chemokine (C-C motif) Ligand 5
CCR5: C-C Chemokine Receptor Type 5
CHOP: the C/EBP Homologous Protein
CM: Conditioned Medium
CXCL10: CXC motif chemokine ligand 10
CXCR2: CXC motif chemokine receptor 2
ER: Endoplasmic Reticulum
IFN-γ: Interferon Gamma
KPC: LSL-KrasG12D/+, LSL-Trp53R172H/+, and Pdx-1-Cre; IL6, interleukin 6
IL12a: Interleukin 12a
LC3: Light Chain 3
MMP9: Matrix Metalloproteinase-9
MPO: Myeloperoxidase
Nectin2: Nectin Cell Adhesion Molecule 2
NK: Natural Killer
OLR1: Oxidized Low Density Lipoprotein Receptor 1
PDAC: Pancreatic Ductal Adenocarcinoma
TAN: Tumor -Associated Neutrophils
TGFβ: Transforming Growth Factor-β
TME: Tumor Microenvironment
TNFα: Tumor Necrosis Factorα
UPR: Unfolded Protein Response
VEGF: Vascular Endothelial Growth Factor
4-PBA: 4-Phenylbutyric acid
