# Supplementary figures and images for: Tumor-associated neutrophils upregulate Nectin2 expression, creating the immunosuppressive microenvironment in pancreatic ductal adenocarcinoma
Source: J Exp Clin Cancer Res. 2024 Sep 11;43:258. doi: 10.1186/s13046-024-03178-6 (PMC11389261; doi:10.1186/s13046-024-03178-6)

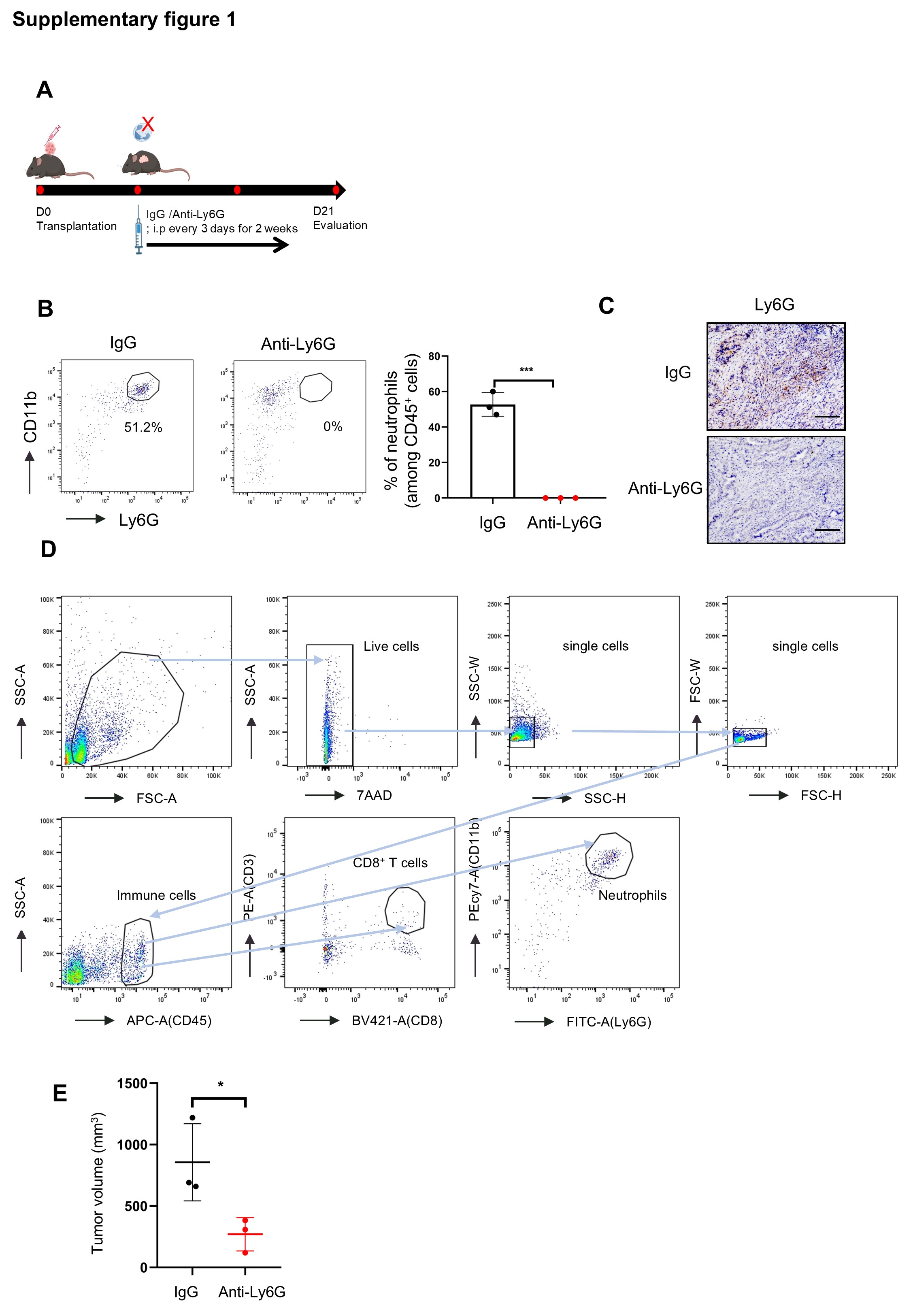

Supplement: Supplementary file 1 — Supplementary Material 1 [file 13046_2024_3178_MOESM1_ESM.jpg]

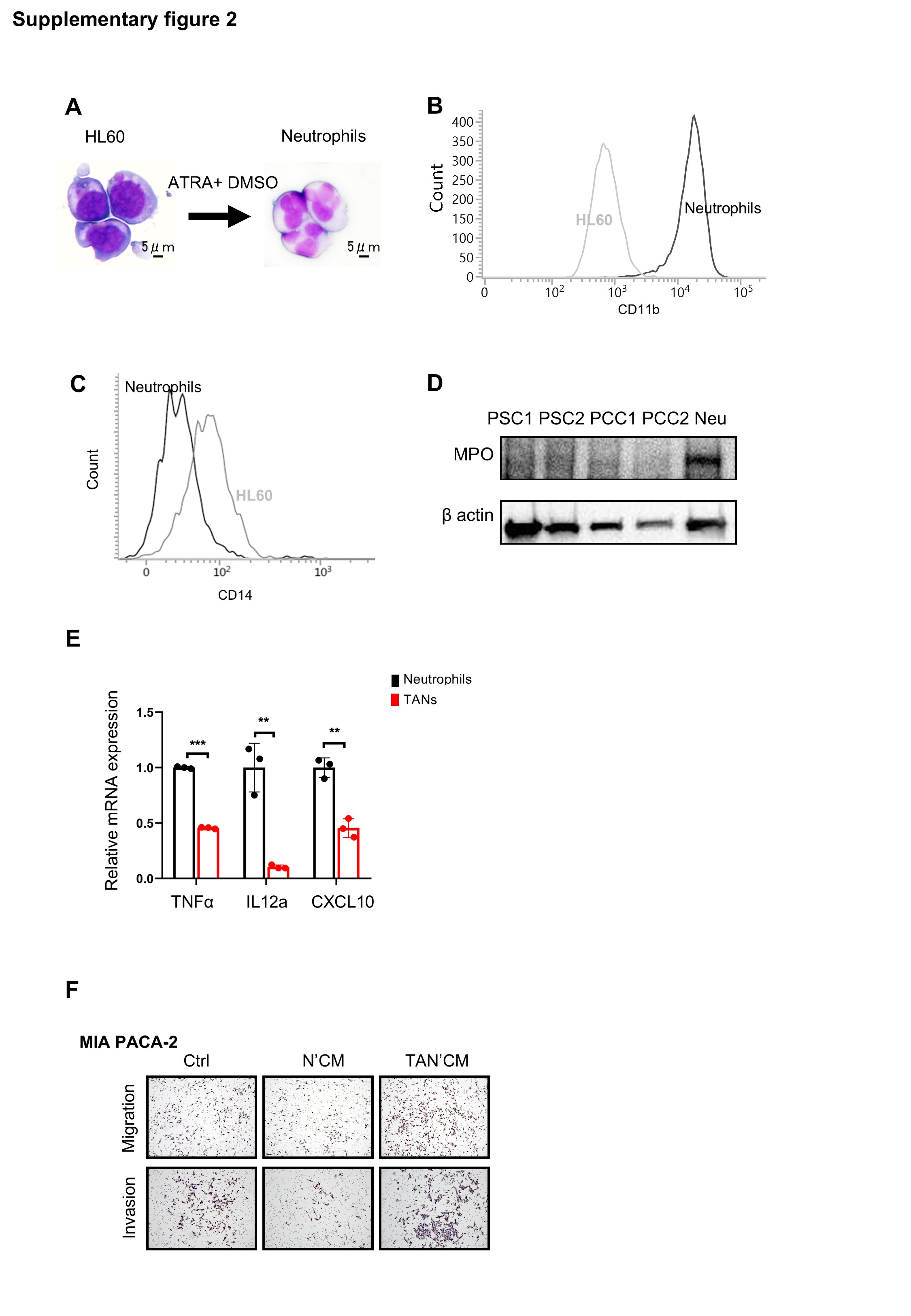

Supplement: Supplementary file 2 — Supplementary Material 2 [file 13046_2024_3178_MOESM2_ESM.jpg]

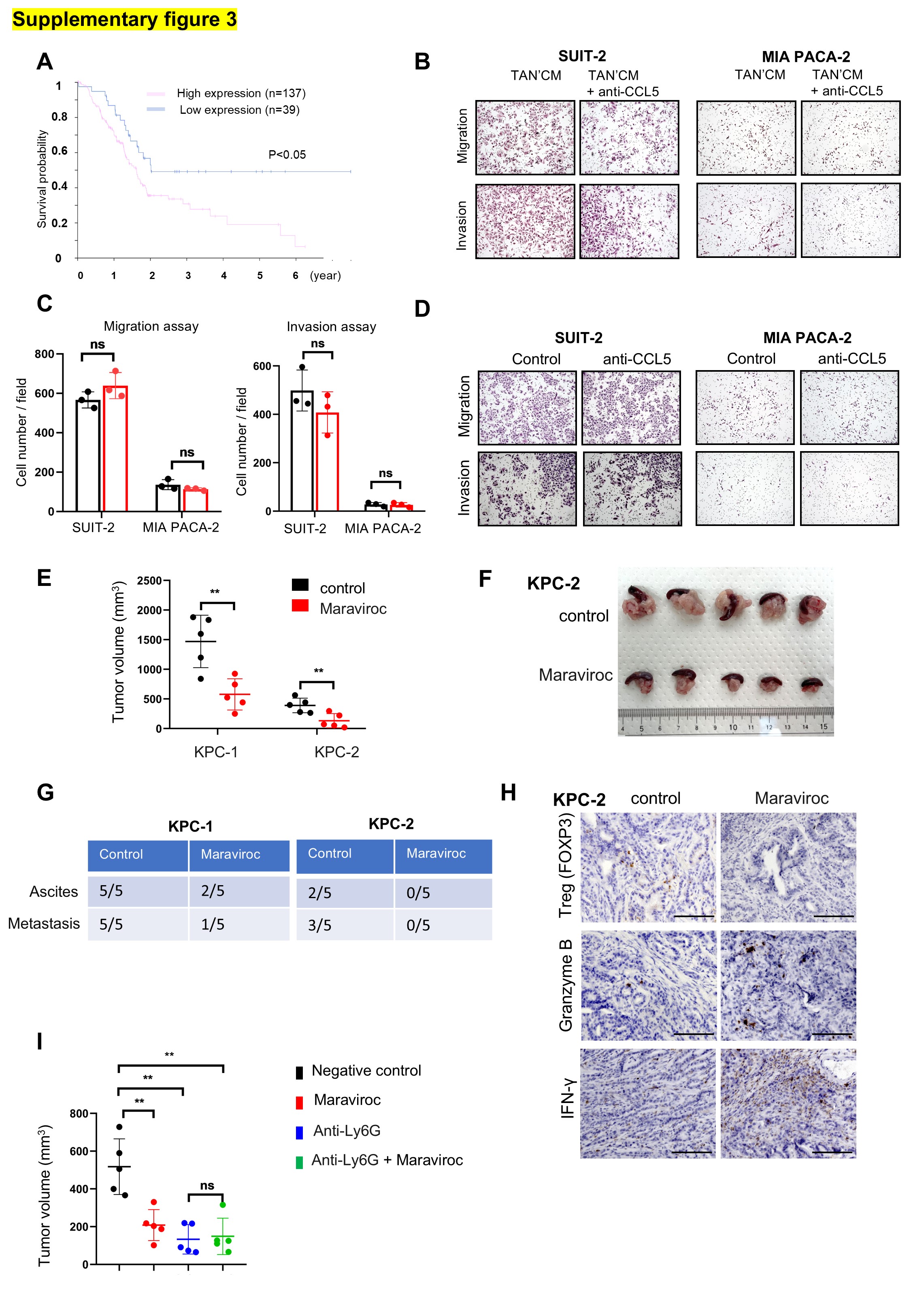

Supplement: Supplementary file 3 — Supplementary Material 3 [file 13046_2024_3178_MOESM3_ESM.jpg]

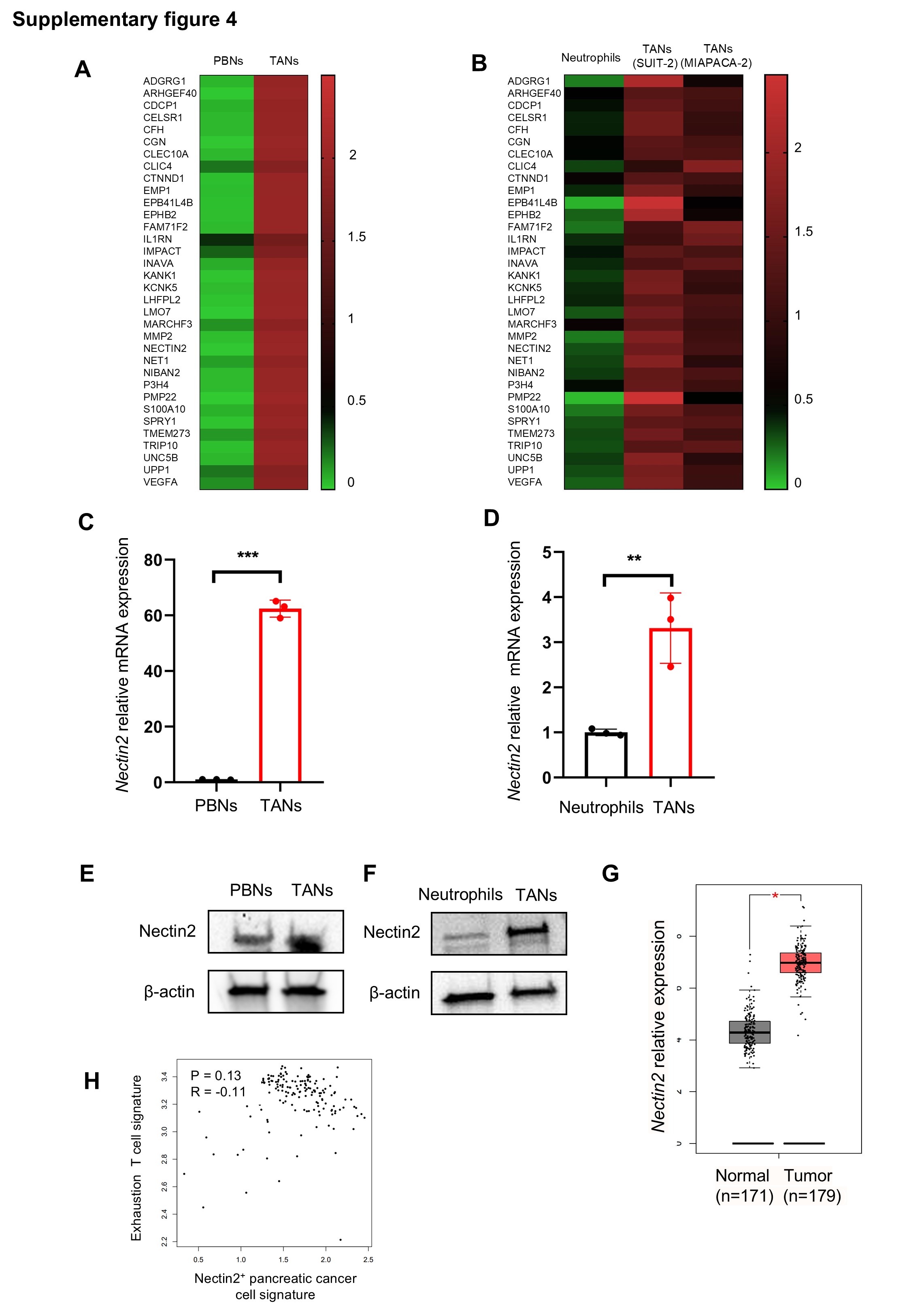

Supplement: Supplementary file 4 — Supplementary Material 4 [file 13046_2024_3178_MOESM4_ESM.jpg]

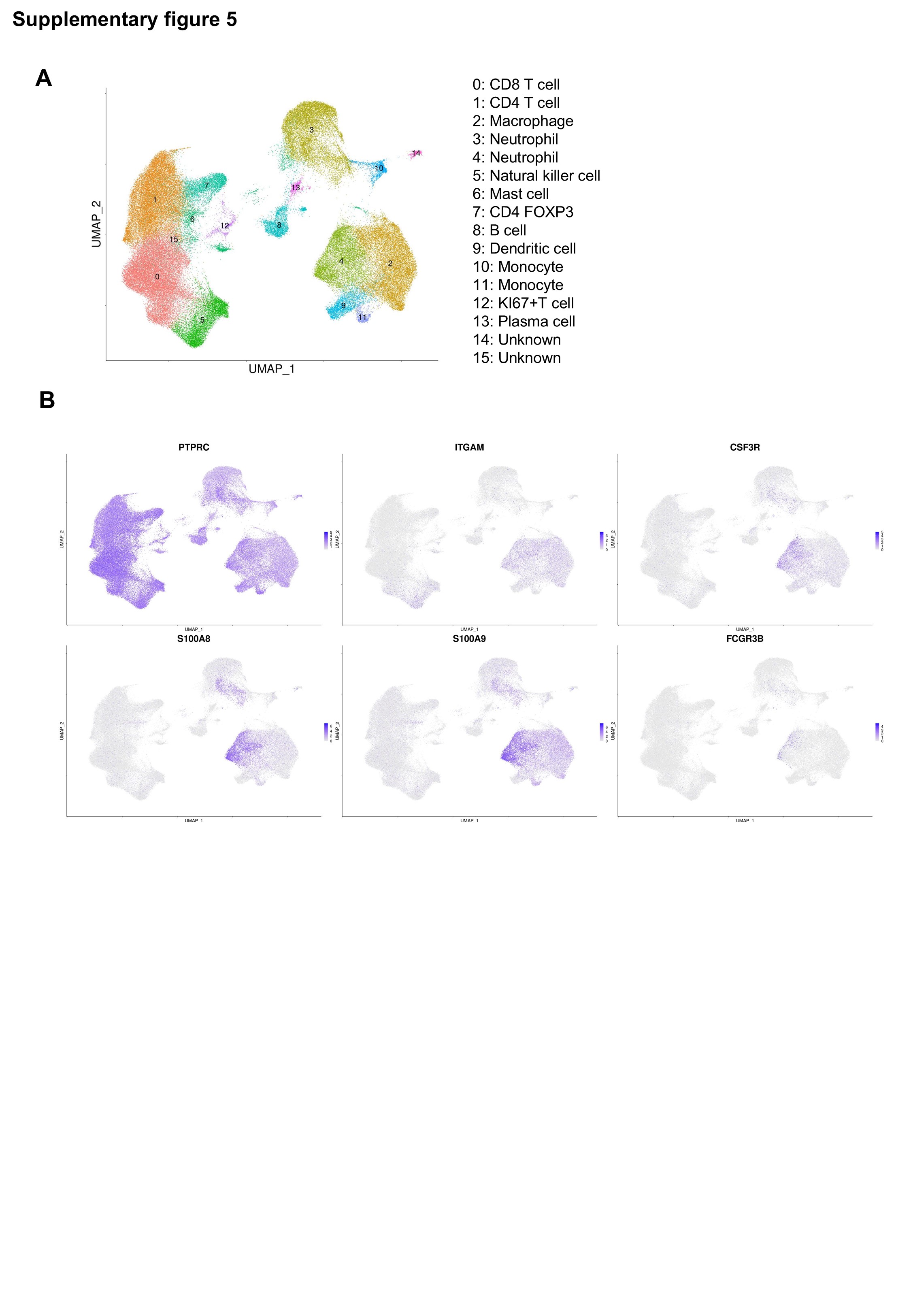

Supplement: Supplementary file 5 — Supplementary Material 5 [file 13046_2024_3178_MOESM5_ESM.jpg]

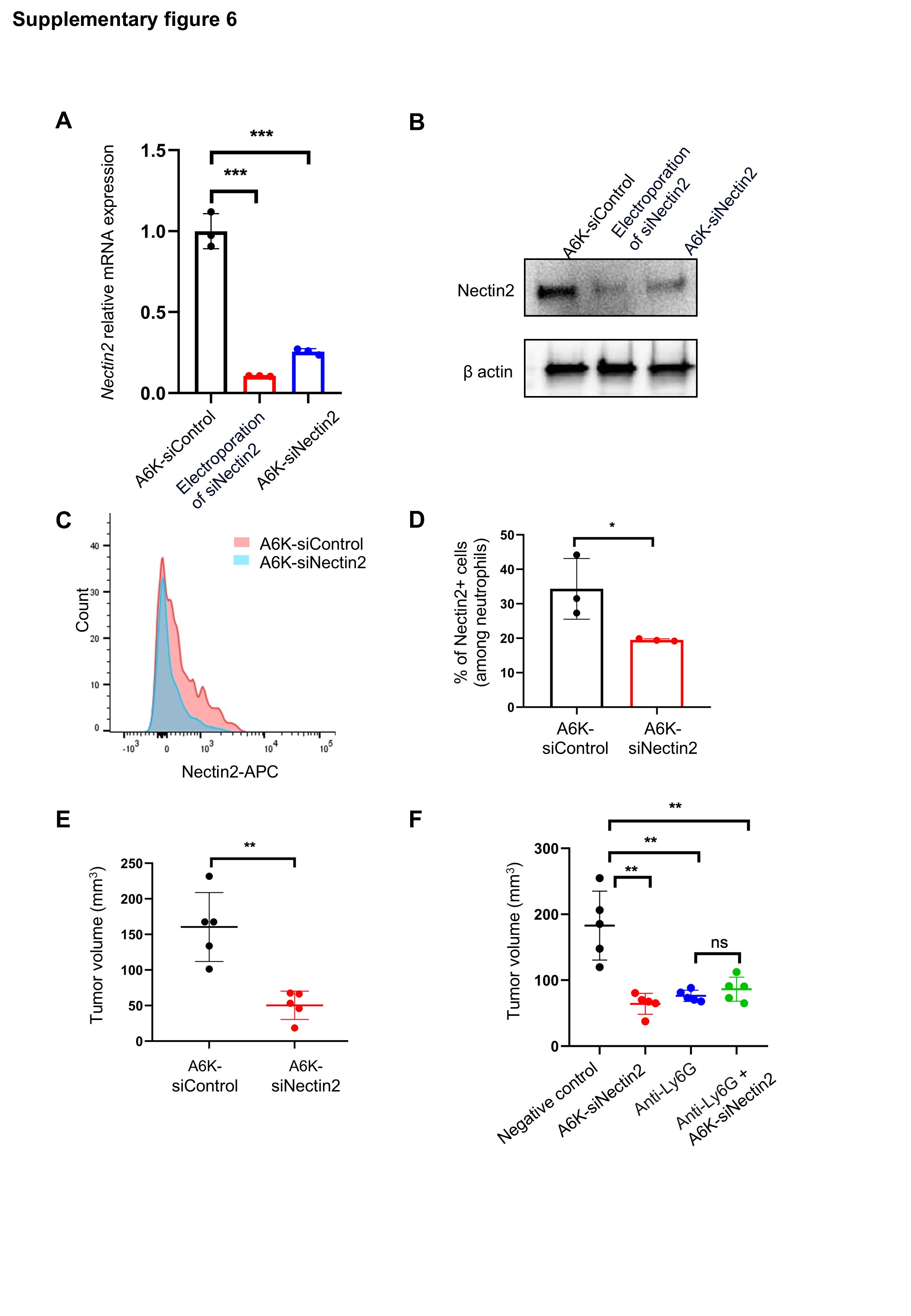

Supplement: Supplementary file 6 — Supplementary Material 6 [file 13046_2024_3178_MOESM6_ESM.jpg]

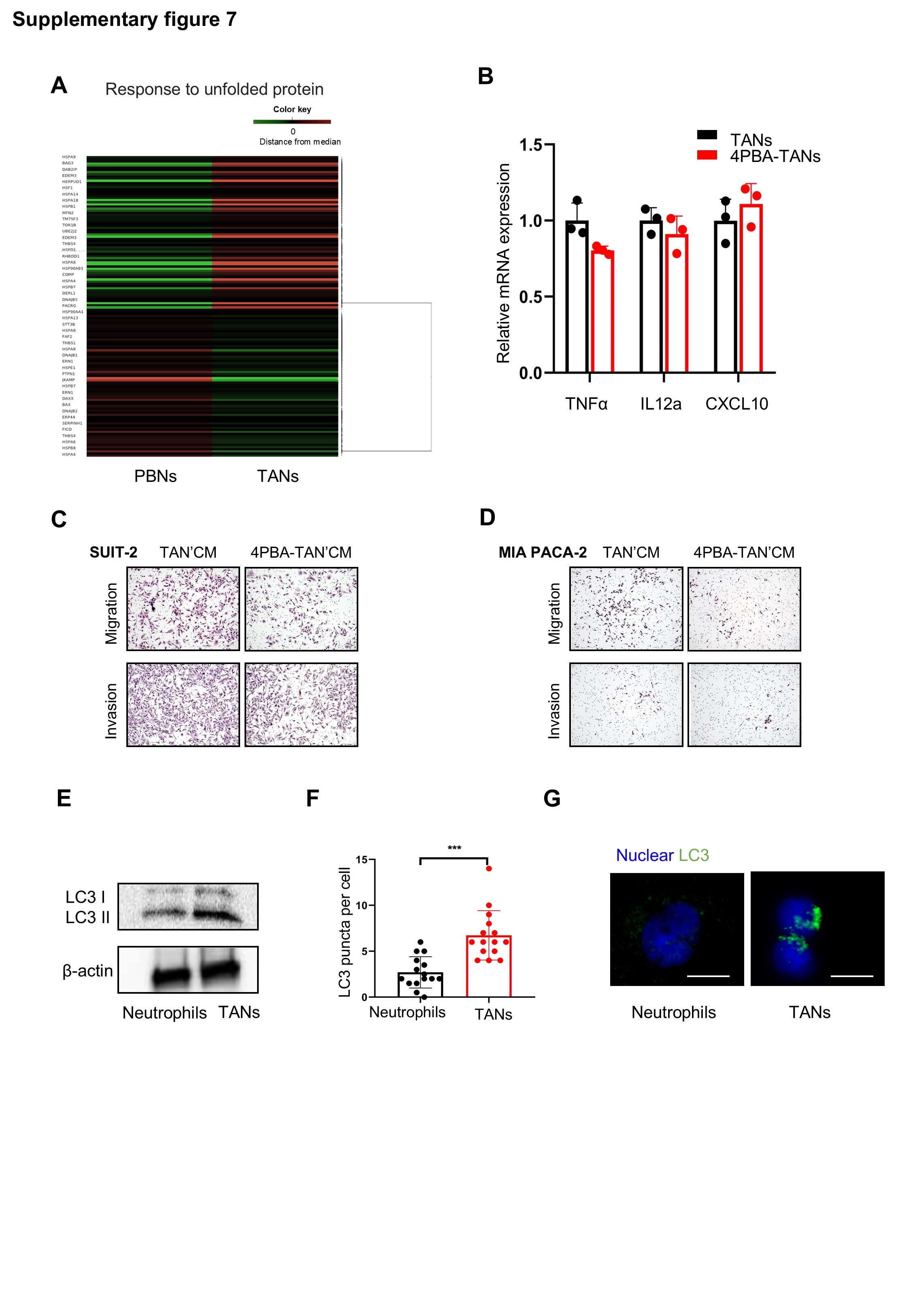

Supplement: Supplementary file 7 — Supplementary Material 7 [file 13046_2024_3178_MOESM7_ESM.jpg]

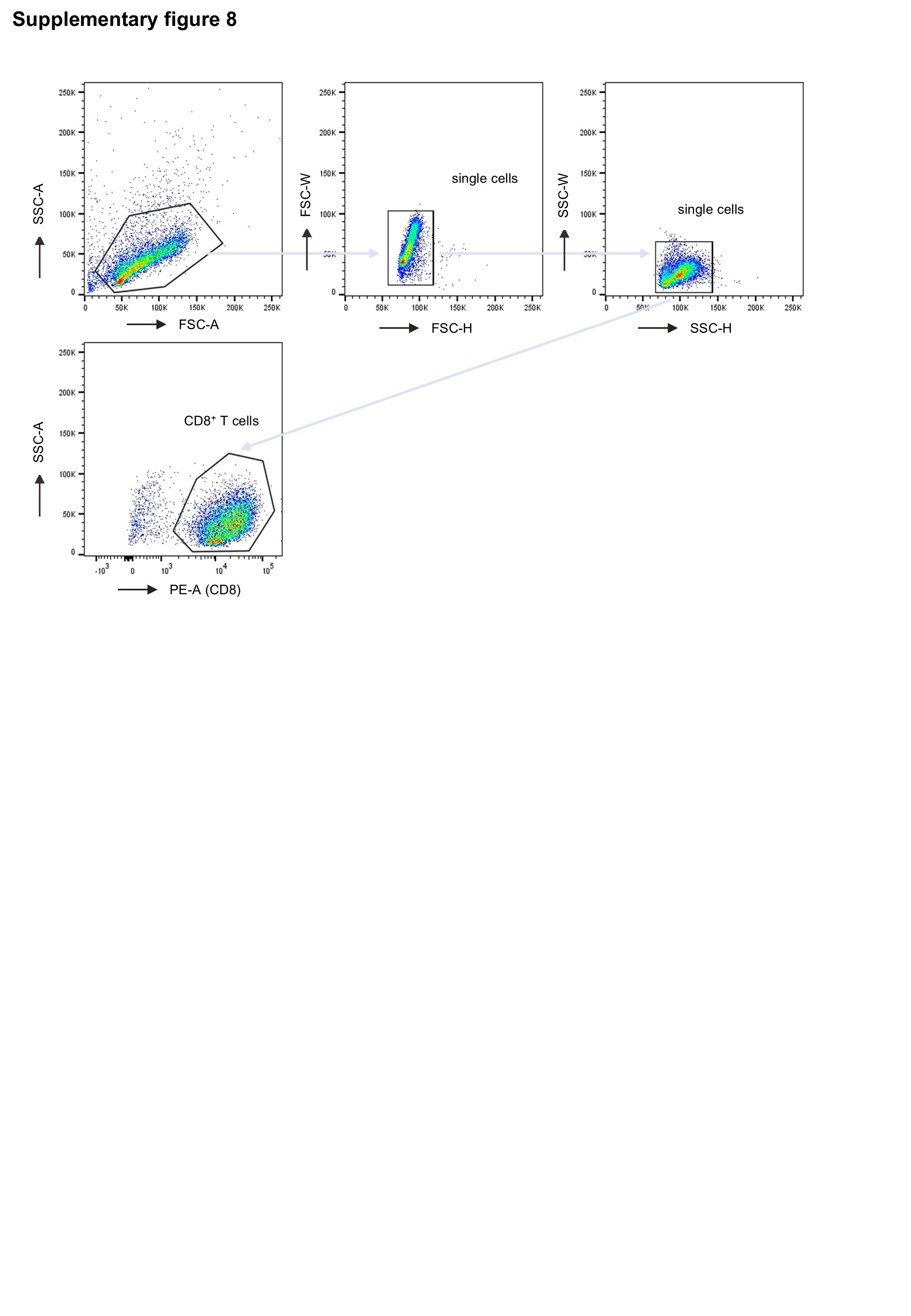

Supplement: Supplementary file 8 — Supplementary Material 8 [file 13046_2024_3178_MOESM8_ESM.jpg]
