# Supplementary material for: Tumor-associated neutrophils upregulate Nectin2 expression, creating the immunosuppressive microenvironment in pancreatic ductal adenocarcinoma
Source: J Exp Clin Cancer Res. 2024 Sep 11;43:258. doi: 10.1186/s13046-024-03178-6 (PMC11389261; doi:10.1186/s13046-024-03178-6)
Supplement: Supplementary file 10 — Supplementary Material 10 [file 13046_2024_3178_MOESM10_ESM.docx]

| Primer | Sequence (5'-3') |
| --- | --- |
| Human |  |
| 18s-F | GTAACCCGTTGAACCCCATT |
| 18s-R | CCATCCAATCGGTAGTAGCCG |
| CD206-F | ACCTCACAAGTATCCACACCATC |
| CD206-R | CTTTCATCACCACACAATCCTC |
| IL6-F | AAGCCAGAGCTGTGCAGATGAGTA |
| IL6-R | TGTCCTGCAGCCACTGGTTC |
| VEGF-F | GAGCCTTGCCTTGCTGCTCTA |
| VEGF-R | CACCAGGGTCTCGATTGGATG |
| MMP9-F | ACCAAGTGGGCTACGTGACCTATG |
| MMP9-R | GTATCCGGCAAACTGGCTCCTT |
| TGFβ-F | GCGACTCGCCAGAGTGGTTA |
| TGFβ-R | GTTGATGTCCACTTGCAGTGTGTTA |
| CXCR2-F | TCTTCAGGGCACACTTCCACTAC |
| CXCR2-R | GGGCTGCATTGACACTGAGA |
| TNFα-F | GCTGCACTTTGGAGTGATCG |
| TNFα-R | TCACTCGGGGTTCGAGAAGA |
| IL12a-F | CCTTGCACTTCTGAAGAGATTGA |
| IL12a-R | ACAGGGCCATCATAAAAGAGGT |
| CXCL10-F | GAAAGCAGTTAGCAAGGAAAGGTC |
| CXCL10-R | ATGTAGGGAAGTGATGGGAGAGG |
| CCL5-F | TCCCACAGGTACCATGAAGGTC |
| CCL5-R | GCAATGTAGGCAAAGCAGCAG |
| Nectin2-F | GAGCACAGCCCACTCAAGAC |
| Nectin2-R | AAGGTGGGCAGCTCATGGTA |
| BIP-F | GAACGTCTGATTGGCGATGC |
| BIP-R | TCAACCACCTTGAACGGCAA |
| CHOP-F | GGAACCTGAGGAGAGAGTGTT |
| CHOP-R | GTCCCGAAGGAGAAAGGCAA |
| ATF6-F | ATGAAGTTGTGTCAGAGAACC |
| ATF6-R | CTCTTTAGCAGAAAATCCTAG |
| Mouse |  |
| GAPDH-F | AATGGTGAAGGTCGGTGTG |
| GAPDH-R | AATGGTGAAGGTCGGTGTG |
| CCL5-F | GAACGTCTGATTGGCGATGC |
| CCL5-R | TCAACCACCTTGAACGGCAA |
| Nectin2-F | AGGCTTCAAGCTGCGGATG |
| Nectin2-R | AGCATCAAAGTATGGCGTCTTCAC |
| BIP-F | CCGAGGAGGAGGACAAGAAG |
| BIP-R | CACATACGACGGCGTGATGC |
| CHOP-F | CCACCACACCTGAAAGCAG |
| CHOP-R | TCCTGCAGATCCTCATACCAG |
| ATF6-F | *TGGGTTCGGATATCGCTGTG* |
| ATF6-R | GGTTCTCTGACACCACCTCG |
