# Supplementary material for: Tumor-associated neutrophils upregulate Nectin2 expression, creating the immunosuppressive microenvironment in pancreatic ductal adenocarcinoma
Source: J Exp Clin Cancer Res. 2024 Sep 11;43:258. doi: 10.1186/s13046-024-03178-6 (PMC11389261; doi:10.1186/s13046-024-03178-6)
Supplement: Supplementary file 11 — Supplementary Material 11 [file 13046_2024_3178_MOESM11_ESM.docx]

**SUPPLEMENTARY MATERIALS**

**METHOD DETAILS**

**Pancreatic adenocarcinoma (PDAC) tissue FFPE sample: immunohistochemistry, immunofluorescence, and evaluation**

Tissues were fixed with formalin, embedded with paraffin, cut into 4-μm thick pieces, and deparaffinized with xylene and ethanol. Endogenous peroxidase activity was blocked using methanol containing 0.3% hydrogen peroxidase. Antigen retrieval was performed in sodium citrate (pH 6.0) or Tris-EDTA (pH 9.0) buffers using a microwave or pressure cooker. Next, the sections were incubated overnight at 4°C with anti-MPO (1:400, #A0398, DAKO) and anti-CD8a (1:1, #423201, Nichirei Biosciences, Japan) primary antibodies for the human sample, as well as anti-CD8 (1:100, #98941; Cell Signaling Technology), anti-granzyme B (1:500, #14-8822-82, Invitrogen), anti-IFNγ (at 1:100, #orb555872, Biorbyt), anti-FOXP3 (1:100, #FJK-16s, eBioscience™), anti-CCL5 (1:20, #710001, Invitrogen), and anti-Ly6G (1:100, #551459, BD bioscience) antibodies for the mouse samples. Afterward, the sections were stained with EnVisiont System-HRP Labeled Polymer Anti-Rabbit (#K4003; Dako) or EnVisiont System-HRP Labeled Polymer Anti-Mouse (#K4001; Dako) antibodies. All staining intensity assessments were performed using the BZ-X Analyzer software (KEYENCE, Osaka, Japan). The number of MPO^+^ cells was evaluated in five representative fields at 100× magnification to analyze neutrophil infiltration, and the average number of MPO^+^ cells per patient was calculated. Sixty patients were equally divided into two groups – high-MPO and low-MPO – based on the neutrophil infiltration level. Serial slides from 31 patient samples were used to determine the correlation between CD8^+^ T-cells and neutrophils. To analyze CCL5, FOXP3, granzyme B, and IFN γ expression in mouse tumors, the positive stained cell or area was measured in three or five fields, and the average was calculated in each sample.

Multiple immunohistochemical (IHC) staining was performed as previously described.[1] Slides were blocked with peroxidase-blocking (#41563138, DAKO) and stained with human: anti-MPO (1:400, #A0398, DAKO), anti-Nectin2 (1:200, #95333T, Cell Signaling Technology), anti-CD8 (1:100, #423201, Nichirei Biosciences), and anti-granzyme B (diluted at 1:100, #SC-8022; Santa Cruz). Each tissue sample was positively stained in five representative fields at 200× magnification using a light microscope (KEYENCE). Seven samples were used to analyze the relationship between Nectin2^+^ neutrophil and granzyme B^+^ CD8^+^ T-cells. Each dot represented one field, and each sample contained five fields.

For immunofluorescence, sections were blocked with 3% bovine serum albumin in phosphate-buffered saline (PBS) and subsequently incubated with anti-MPO (7 µg/mL, #AF3667, R&D System) and anti-nectin2 (1:100, #ab135246, Abcam). Nuclei were counterstained with hematoxylin or 4, 6-diamidino-2-phenylindole (DAPI; Dojindo, Kumamoto, Japan). The stained area was measured in three fields for each sample at 100× magnification using a fluorescence microscope. Eleven samples were used to evaluate Nectin2^+^ neutrophils in the tumor area and adjacent normal pancreatic tissues.

**Mouse experiments**

Four-week-old C57BL/6 female mice purchased from CLEA Inc. (Tokyo, Japan) were used as syngeneic PDAC models. To establish orthotopic models, the mice were implanted with KPC cells (KPC-1: 1 × 10^5^; KPC-2: 5 × 10^5^) suspended in 50 µL of Dulbecco’s modified Eagle’s medium (DMEM; Sigma-Aldrich, St. Louis, MO, USA). For subcutaneous models, KPC cells (KPC-1: 3 × 10^5^; KPC-2: 7 × 10^5^) suspended in 50 µL of DMEM were implanted. All cell lines tested negative for *Mycoplasma* contamination before each animal experiment. One week after the implantation, the mice were injected with maraviroc (a CCR5 inhibitor, 15 mg/kg, intraperitoneal; HY-13004/ PZ0002, MCE/sigma) thrice weekly for 2 weeks or siRNA-A6K (0.05 mL/mouse, intratumoral) every 3 days for 2 weeks. Neutrophils were depleted by intraperitoneal injection of anti-Ly6G (clone 1A8; BioXCell; Cat# BE0075-1) or isotype control antibody (clone 2A3, BioXcell; Cat# BP0089) diluted in InVivoPure pH 7.0 dilution buffer (BioXcell; Cat # IP0070) at an initial dose of 400 μg, followed by 100 μg every 3 days for 2 weeks. The tumor volume was calculated using the following formula: π/6 × (length × width × width).

All animal experimental protocols were approved by the Ethics Committee of Kyushu University (A22-128; A22-160; A22-161; A22-339) and performed following the guidelines of the Institutional Animal Committee of Kyushu University.

**Western blotting**

The total proteins were extracted using PRO-PREP Protein Extraction Solution (#17081, iNtRON Biotechnology). Cytosolic and membrane proteins were extracted using the Mem-PER™ Plus Membrane Protein Extraction Kit (#89842; Thermo Fisher Scientific) according to the manufacturer’s instructions. The protein concentration was normalized to 20 µg/40 µL per sample. Samples were boiled at 95 °C for 10 min, loaded to 4–15% Mini-PROTEAN TGX Precast Gels (#456-1084, Bio-Rad Laboratories), and transferred to Trans-Blot Turbo Mini PVDF Transfer Packs (#170-4156, Bio-Rad Laboratories) using a Trans-Blot Turbo Transfer Starter System (Bio-Rad Laboratories). Then, membranes were blocked and incubated with anti-nectin2 (1:1000, #135246, Abcam), anti-BHLHE40 (1:1000, #B100-1800. Novus biologicals), anti-BIP (1:1000, #3177, Cell Signaling Technology), anti-CHOP (1:1000, #2895, Cell Signaling Technology), anti-pERK (1:1000, #5683S, Cell Signaling Technology), anti-LC3 (1:1000, #2775, Cell Signaling Technology), mouse anti-β-actin (1:1000, #ab8227; Abcam), and rabbit anti-β-actin (1:1000, #4970S; Cell Signaling Technology) at 4°C overnight. After washing thrice with Tris Buffered Saline with Tween-20 (TBST) buffer, the membranes were probed with horseradish peroxidase-conjugated secondary antibodies. Immunoblotting was performed using a ChemiDoc XRS System (Bio-Rad Laboratories) and analyzed using Quantity One Software (Bio-Rad Laboratories).

**Flow cytometry**

HL-60 cells were washed and suspended in 100 µL PBS. Then, they were incubated with FITC-conjugated CD11b (ICRF44, #562793, BD Bioscience), FITC-conjugated CD14 (M5E2, # 561712, BD Bioscience), PE-conjugated Nectin2 (R2.525, # 551057, BD Bioscience) for 30 min at 4°C. After removing the antibodies with PBS, the cells were stained with propidium iodide (1:1000, #421301; BioLegend). For mouse samples, fresh murine PDAC tumors were sliced into 0.5–1.0 mm fragments and dissolved using the Tumor Dissociation Kit (#130-960-730, Miltenyi Biotec) according to the manufacturer’s instructions. Next, 1 × 10^6^ cells were suspended in 100 µL PBS and preincubated with anti-mouse CD16/CD32 antibody (BD Bioscience) for 10 min to prevent non-specific binding via the Fcγ receptor. Subsequently, the samples were incubated with the following antibodies at 4°C: APC-conjugated CD45 antibodies (30-F11, #103112, BioLegend), BV421-conjugated CD45 (30-F11, #563890, BioLegend), PE-cy7-conjugated CD11b (M1/70, #552850, BD Pharmingen), PE-conjugated antibodies to mouse CD3 (17A2, #100206, BioLegend), BV421-conjugated antibodies to mouse CD8α (53-6.7, #553031, BD Horizon), PE-conjugated antibodies to mouse CD8α (53-6.7, #553032, BD Pharmingen), FITC-conjugated antibodies to mouse Ly-6G (1A8, #127605, BioLegend), APC-conjugated antibodies to Nectin2 (829038, #FAB3869A, R&D system), APC-conjugated Granzyme B (QA16A02, #372204, BioLegend), AF700-conjugated IFN γ (XMG1.2, #557998, BD Pharmingen), APC-conjugated PD1 (J43, #562673, BD Pharmingen), and BV480-conjugated TIM3 (5D12, #747618, BD Biosciences). After 30 min, the samples were washed and suspended in PBS containing 7-amino-actinomycin D (BD Horizon, 559925) for subsequent experiments. The cells were analyzed using a FACSAria flow cytometer (BD Biosciences). In each experiment, single-stained samples were prepared for each antibody to determine the appropriate voltage and compensation. The gating strategies for flow cytometry analysis are shown in online supplemental figures 1D and 9. Data analysis was performed using Flow Jo 10.5.3 (BD Biosciences) with appropriate compensation.

**Quantitative reverse transcription polymerase chain reaction (****qRT-PCR)**

In this study, qRT-PCR was performed using the iTaq Universal SYBR Green One-Step kit and the CFX96 Touch Real-Time PCR Detection system (Bio-Rad Laboratories). Total RNA was extracted using a High Pure RNA Isolation Kit (#11828665001, Roche, Basel, Switzerland) according to the manufacturer’s instructions. The primers were purchased from Takara Bio (Shiga, Japan) and Sigma-Aldrich. The primers used for the target genes are listed in the online supplemental Table 2. *GAPDH* and *18S* were used as endogenous controls. The samples were analyzed in triplicates.

According to previous reports,[2-6] *CD206*, *IL6*, *VEGF*, *MMP9*, *CXCR2*, and *TGFβ* are recognized as N2 marker genes, and *TNFα*, *IL12a,* and *CXCL10* are recognized as N1 marker genes.

**Small interfering RNA silencing (siRNA) and siRNA complex preparation**

The siRNA and A6K (gift from 3-D Matrix, Tokyo, Japan) complexes were generated as described previously.[7] Briefly, 100 nM siRNA for *Nectin2* (siNectin2; GS19294, Qiagen) or negative control (siControl; #1027310) was mixed with 50 µM A6K solution using the sonic rotator at 10 rpm for 20 min at room temperature. The generated A6K-siRNA complex was transfected into cells through direct incubation for 72 h. For the *in vivo* experiment, siRNA and A6K solution were mixed to a final concentration of 8.1/0.038 mM A6K/siRNA. The A6K-siRNA complex solution (100 μL) was injected into the subcutaneous tumors of mice every 3 days for 2 weeks. KPC-1 cells were directly mixed with A6K-siControl siNectin2 or A6K-siNectin2 to check knockdown efficiency. As a positive control for transfection, electroporation was performed using a Nucleofector System (Lonza, Basel, Switzerland) following the manufacturer’s instructions. Cells were cultured for 72 h after transfection and collected for PCR and western blot analyses.

**Isolation of mouse neutrophils**

For tumor-associated neutrophils, single-cell suspensions were prepared as described under ‘Flow cytometry.’ For peripheral blood-derived neutrophils, whole blood was treated with ACK lysis buffer (#1049201, Thermo Fisher) to remove red blood cells. A Mouse Ly6G Selection Kit (BioLegend, #480124) was used to isolate neutrophils according to the manufacturer’s protocol.

**Neutrophils and CD8^+^ - cell co-culture**

CD8^+^ T-cells were isolated from the spleens of tumor-bearing mice using a CD8^+^ T-cell Isolation Kit (#130-104-075; Miltenyi Biotec). Magnetic bead-purified peripheral CD8^+^ T-cells (2 × 10^5^ cells/well in 96-well plates) were co-cultured with neutrophils isolated from the tumor or peripheral blood at a 1:10 (T-cell: neutrophil) ratio in a 200 μL solution containing, anti-CD3 (2 μg/mL), and anti-CD28 (2 μg/mL) antibodies, with or without anti-mouse Nectin2 antibody[8] (#MAB3869, R&D System). CD8^+^ T-cells were analyzed using flow cytometry to determine the expression of cytotoxic and exhaustion markers.

**Public data and analysis**

The Cancer Genome Atlas (TCGA) data and Genotype-Tissue Expression project databases were obtained from PDAC tumor tissues. The mRNA expression levels of *CCL5* and *NECTIN2* were analyzed in samples from patients with PDAC and non-tumorigenic controls. The results were generated using the TCGA Research Network (https://www.cancer.gov/tcga). The Gene Expression Profiling Interactive Analysis (GEPIA) tool (http://gepia2.cancer-pku.cn/#analysis) was used to evaluate the relationships of *CCL5* mRNA levels, neutrophil signature (*S100A9*, *S100A8*, and *CSF3R*), and T-cell exhaustion with Nectin2^+^ neutrophils (*CD112*, *S100A9*, *S100A8*, and *CSF3R*) and pancreatic cancer cells (*EPCAM, MUC1, KRT19, CK19, CD112*) in patients with PDAC. The Human Protein ATLAS website (https://www.proteinatlas.org/) was used to evaluate the prognosis of PDAC characterized by CCL5 in patients. Functional and pathway enrichment analyses were performed using Gene Ontology within the Database for Annotation, Visualization, and Integrated Discovery.

**SUPPLEMENTAL FIGURES AND LEGENDS**

**Supplemental figure 1. Neutrophil depletion inhibited tumor progression in PADC, related to Figure 1.** (A) Experimental schema for the neutrophil depletion experiment. (B) Representative flow cytometry image of CD11b and Ly6G in CD45+ cells in the tumor (left). Quantification of CD11b+ Ly6G+ cells among CD45+ cells (right). (C) Immunohistochemistry of Ly6G demonstrating diminished neutrophil infiltration into the TME. Scale bar, 100 µm. (D) The gating strategy used during flow cytometry for neutrophils and CD8^+^ T-cells in the neutrophil depletion experiment. (E) Representative quantified graph of tumor volume in neutrophil-depleted mice. *p < 0.05, ***p < 0.001.

**Supplemental figure 2. Generation of TANs from human progenitor cells. TANs promoted pancreatic cancer cell migration and invasion, related to Figure 1.** (A) Giemsa staining of HL60 cells showing round nuclei and neutrophils differentiated from HL60 cells with ATRA and DMSO showing the typical segmented nuclei. Scale bar, 5 µm. (B and C) CD11b (B) and CD14 (C) expression, as assessed using flow cytometry in HL-60 (gray) and generated human neutrophils (black). (D) The MPO expression assessed by western blot in cultured pancreatic stellate cells (PSC), pancreatic cancer cells (PCC) and generated neutrophils (Neu). (E) Quantification of N1 marker genes in neutrophils differentiated from HL60 and those treated with the supernatant of SUIT-2 cells (TANs). *18S* served as an internal control (*n* = 3 per group). (F) Representative microphotographs of migrating (top) and invading (bottom) MIA PACA-2 cells treated with a conditioned medium of neutrophils (N’CM) or TANs (TAN’CM). **p < 0.01, ***p < 0.001.

**Supplemental figure 3. TANs promote tumor progression through the CCL5–CCR5 axis, related to Figure 2.** (A) Kaplan–Meier survival analysis of the TCGA data of patients with PDAC characterized by high (red line) and low (blue line) CCL5 expression. (B) Representative microphotographs of migrating (top) and invading (bottom) SUIT-2 and MIA PACA-2 cells with or without anti-CCL5 antibody. TAN’CM indicates the conditioned medium of TANs. (C-D) Migration and invasion assay of SUIT-2 or MIA PACA-2 cells without treatment of TANs conditioned medium. (n = 3 per group). Quantification (C) and representative microphotographs (D). (E) A quantified graph of tumor volume for mice orthotopically transplanted with KPC-2 cells. (F) Representative *in situ* images of tumors for KPC-2-transplanted mice. (G) The number of KPC-1- or KPC-2-transplanted mice with ascites and tumor dissemination. (H) Representative immunohistochemical staining of FOXP3^+^, Granzyme B^+^, and IFNγ^+^ cells in KPC-2-transplanted tumors. Scale bar, 100 μm. (I) A quantified graph of tumor volume for orthotopically transplanted mice with KPC-2 cells after treatment with maraviroc and anti-Ly6G. *p < 0.05, **p < 0.01; ns, not significant.

**Supplemental figure 4. TANs upregulated Nectin2 expression in humans and mice, related to Figure 4.** (A and B) Heatmap of 34 genes whose expression is universally upregulated in mouse (A) and human (B) TANs. (A) Neutrophils from peripheral blood (PBNs) and those in KPC-1 tumors (TANs) from orthotopically transplanted mice analyzed through microarrays. (B) Gene expression in HL60-derived human neutrophils polarized with SUIT-2 or MIAPACA-2 supernatants. (C) The relative expression levels of *Nectin2* in PBNs and TANs from orthotopically implanted pancreatic tumors in mice. The expression level of *GAPDH* served as an internal control (*n* = 3 per group). (D) The relative expression level of *Nectin2* in HL60 cell-derived neutrophils and neutrophils treated with the supernatant of cancer cells (TANs). The expression level of *18S* served as an internal control (*n* = 3 per group). (E) The expression levels of Nectin2 in PBNs and TANs from mice quantified through western blotting. (F) The expression levels of Nectin2 in HL60 cell-derived neutrophils and TANs treated with the supernatant of cancer cells (TANs), as quantified through western blotting. In this analysis, the western blot membrane used in **Figure 6C** was re-probed with the anti-Nectin2 antibody. Accordingly, the same image of β-actin band shown in **Figure 6C** is repeatedly presented as a reference. (G) Gene expression profiling interactive analysis of *Nectin2* in normal (gray bar) and tumor (red bar) samples from PDAC data in The Cancer Genome Atlas (TCGA) and Genotype-Tissue Expression databases. (H) Pearson correlation analysis of the mRNA levels in Nectin2+ pancreatic cancer cells (*CD112, EPCAM MUC1, KRT19, CK19*) and exhausted T-cell (*HAVCR2, TIGIT, LAG3, PDCD1, CXCL13, LAYN*) signatures in 177 human PDAC from the TCGA database. *p < 0.05, **p < 0.01, ***p < 0.001.

**Supplemental figure 5. Single-cell analysis for neutrophils in PDAC, related to Figure 4.** (A) Analysis of the scRNA-seq dataset GSE205013 of human PDAC. Uniform manifold approximation and projection of total CD45+ cells segregated into 15 clusters of main immune populations as described previously.[9] (B) Neutrophils identified as Clusters 3 and 4 based on the expression of neutrophil markers (*S100A8, S100A9, CSF3R*, and *FCGR3B*)[10] and re-clustered using uniform manifold approximation and projection.

**Supplemental figure 6. Inhibition of Nectin2 expression by the A6K-siNectin2 complex, related to Figure 5.** (A-B) KPC-2 cells were subjected to Nectin2 knockdown experiments. Quantitative qRT-PCR assays (A) and western blotting (B) verified the efficacy of Nectin2 knockdown by A6K-siNecin2. Electroporation of siNectine2 served as a positive control in the western blot. (C-D) Representative flow cytometry analysis of Nectin2 expression in neutrophils isolated from subcutaneous KPC-1-transplanted tumors. (E) A quantified graph of tumor volume for mice subcutaneously implanted with KPC-2 cells. (F) A quantified graph showing tumor volume after anti-Ly6G and A6K-siNectin2 treatment. *p < 0.05, **p < 0.01, ***p < 0.001.

**Supplemental figure 7. ER stress increased in TANs, producing their pro-tumor functions, related to Figure 6.** (A) heatmap for the genes involved in the response to unfolded proteins. (B) Relative expression levels of N1 marker genes in human (TANs) and 4-Phenylbutyric acid (4-PBA)-pretreated TANs (4PBA-TANs). The mRNA expression level of each gene was normalized to fold over 18S. (n = 3 per group). (C-D) Representative microphotographs of migrating (top) and invading (bottom) SUIT-2 (C) and MIA PACA-2 (D) cells with TAN’CM or 4PBA-TAN’CM. (E) Western blotting results demonstrate LC3II expression in HL60 cell-derived neutrophils and those treated with the supernatant of SUIT-2 cells (TANs). β-actin was used as a loading control. (F-G). The quantification (F) and representative image (G) of immunofluorescence staining of LC3 in human neutrophils and TANs. Scale bar, 10 μm.

**Supplemental figure 8. Gating strategy used for CD8+ T cell co-culture in flow cytometry, related to Figure 3.** Gating stratagem of flow cytometry for CD8+ T cells in the co-culture system.

**Supplemental table 1.** Statistical data of four clusters of tumor-associated neutrophils in human PDAC.

**Supplemental table 2.** Primers used for qRT-PCR

**References**

1. Tsujikawa T, Kumar S, Borkar RN, Azimi V, Thibault G, Chang YH*, et al.* Quantitative multiplex immunohistochemistry reveals myeloid-inflamed tumor-immune complexity associated with poor prognosis. Cell reports 2017;19:203-17

2. Qi M, Xia Y, Wu Y, Zhang Z, Wang X, Lu L*, et al.* Lin28B-high breast cancer cells promote immune suppression in the lung pre-metastatic niche via exosomes and support cancer progression. Nat Commun 2022;13:897

3. Chao T, Furth EE, Vonderheide RH. CXCR2-dependent accumulation of tumor-associated neutrophils regulates T-cell immunity in pancreatic ductal adenocarcinoma. Cancer immunology research 2016;4:968-82

4. Bekes EM, Schweighofer B, Kupriyanova TA, Zajac E, Ardi VC, Quigley JP, Deryugina EI. Tumor-recruited neutrophils and neutrophil TIMP-free MMP-9 regulate coordinately the levels of tumor angiogenesis and efficiency of malignant cell intravasation. The American journal of pathology 2011;179:1455-70

5. Liang W, Ferrara N. The complex role of neutrophils in tumor angiogenesis and metastasis. Cancer immunology research 2016;4:83-91

6. Shaul ME, Levy L, Sun J, Mishalian I, Singhal S, Kapoor V*, et al.* Tumor-associated neutrophils display a distinct N1 profile following TGFβ modulation: A transcriptomics analysis of pro-vs. antitumor TANs. Oncoimmunology 2016;5:e1232221

7. Yoshida D, Kim K, Takumi I, Yamaguchi F, Adachi K, Teramoto A. A transfection method for short interfering RNA with the lipid-like self-assembling nanotube, A6K. Medical molecular morphology 2013;46:86-91

8. Ho DW-H, Tsui Y-M, Chan L-K, Sze KM-F, Zhang X, Cheu JW-S*, et al.* Single-cell RNA sequencing shows the immunosuppressive landscape and tumor heterogeneity of HBV-associated hepatocellular carcinoma. Nat Commun 2021;12:3684

9. Okuda S, Ohuchida K, Nakamura S, Tsutsumi C, Hisano K, Mochida Y*, et al.* Neoadjuvant chemotherapy enhances anti-tumor immune response of tumor microenvironment in human esophageal squamous cell carcinoma. Iscience 2023;26

10. Xue R, Zhang Q, Cao Q, Kong R, Xiang X, Liu H*, et al.* Liver tumour immune microenvironment subtypes and neutrophil heterogeneity. Nature 2022;612:141-7
